# High resolution mapping of nitrogen dioxide and particulate matter in Great Britain (2003–2021) with multi-stage data reconstruction and ensemble machine learning methods

**DOI:** 10.1016/j.apr.2024.102284

**Published:** 2024-08-09

**Authors:** Arturo de la Cruz Libardi, Pierre Masselot, Rochelle Schneider, Emily Nightingale, Ai Milojevic, Jacopo Vanoli, Malcolm N. Mistry, Antonio Gasparrini

**Affiliations:** a Environment & Health Modelling (EHM) Lab, Department of Public Health Environments and Society, London School of Hygiene & Tropical Medicine, 15-17 Tavistock Place, WC1H 9SH, London, United Kingdom; b Department of Public Health, Environments and Society, London School of Hygiene & Tropical Medicine, 15-17 Tavistock Place, WC1H 9SH, London, United Kingdom; c Department of Infectious Disease Epidemiology and Dynamics, London School of Hygiene & Tropical Medicine, Keppel Street, WC1E 7HT, London, United Kingdom; d Centre on Climate Change & Planetary Health, London School of Hygiene & Tropical Medicine, Keppel Street, WC1E 7HT, London, United Kingdom; e Φ-lab (Phi-lab), European Space Agency (ESA), Frascati, Italy; f Forecast Department, European Centre for Medium-Range Weather Forecast (ECMWF), Reading, United Kingdom; g School of Tropical Medicine and Global Health, Nagasaki University, Nagasaki, Japan; h Department of Economics, Ca’ Foscari University of Venice, Italy

**Keywords:** Air pollution, Nitrogen dioxide, Particulate matter, Machine learning, ensemble GIS

## Abstract

In this contribution, we applied a multi-stage machine learning (ML) framework to map daily values of nitrogen dioxide (NO_2_) and particulate matter (PM_10_ and PM_2.5_) at a 1 km^2^ resolution over Great Britain for the period 2003–2021. The process combined ground monitoring observations, satellite-derived products, climate reanalyses and chemical transport model datasets, and traffic and land-use data. Each feature was harmonized to 1 km resolution and extracted at monitoring sites. Models used single and ensemble-based algorithms featuring random forests (RF), extreme gradient boosting (XGB), light gradient boosting machine (LGBM), as well as lasso and ridge regression. The various stages focused on augmenting PM_2.5_ using co-occurring PM_10_ values, gap-filling aerosol optical depth and columnar NO_2_ data obtained from satellite instruments, and finally the training of an ensemble model and the prediction of daily values across the whole geographical domain (2003–2021). Results show a good ensemble model performance, calculated through a ten-fold monitor-based cross-validation procedure, with an average R^2^ of 0.690 (range 0.611–0.792) for NO_2_, 0.704 (0.609–0.786) for PM_10_, and 0.802 (0.746–0.888) for PM_2.5_. Reconstructed pollution levels decreased markedly within the study period, with a stronger reduction in the latter eight years. The pollutants exhibited different spatial patterns, while NO_2_ rose in close proximity to high-traffic areas, PM demonstrated variation at a larger scale. The resulting 1 km^2^ spatially resolved daily datasets allow for linkage with health data across Great Britain over nearly two decades, thus contributing to extensive, extended, and detailed research on the long-and short-term health effects of air pollution.

## 1 Introduction

Air pollution presents a threat to human health, with acute or long-term exposure to several pollutants linked to increased health risks (WHO, 2016). For instance, particulate matter, both coarse (PM_10_) and fine (PM_2.5_) components, as well as gaseous pollutants such as nitrogen dioxide (NO_2_), are independently associated with increased mortality and morbidity (Huangfu and Atkinson, 2020; Liu et al., 2019; Mills et al., 2015, 2016). Epidemiological analyses of health risks of these pollutants necessitate accurate exposure measurements across large populations, commonly reconstructed through air pollution models. Traditionally, these are produced through various methods, such as land-use regression (LUR), emission-dispersion models (EDMs), atmospheric reanalysis, or remote-sensing satellite measurements, with the choice dependent on the study settings and aims, data geospatial coverage, and the geographical and temporal domains of the analysis.

Traditional LUR methods combine land-use information with demographic and ancillary data, offering good performance at reduced geographic scales (Ryan and LeMasters, 2007). However, they are less effective for modelling pollutant levels across large areas, or for detecting short-term temporal patterns, due to sparse and spatially unbalanced monitoring points, as well as the limited resolution of predictor data (Hoek et al., 2008). In contrast, emission-dispersion models (EDMs) utilise high-powered computing to simulate the physical transport of pollutants emitted from known sources and gathered in reliable inventories (Johnson, 2022). They are suitable for modelling air pollution on large geographical scales (Ge et al., 2021; Holmes and Morawska, 2006) and are commonly used in multi-city, regional, and global impact evaluations (Anenberg et al., 2019; Orru et al., 2022; Rittner et al., 2020). Alternatively, atmospheric composition reanalysis such as that performed by the European Centre for Medium-Range Weather Forecasts (ECMWF) (Inness et al., 2019), assimilate a large number of sources to produce gap-less products covering very wide geographical domains. However, the predictive ability of EDMs and reanalyses is generally limited, and their resolution, particularly that of reanalysis data, may not be fine enough for reconstructing high-quality exposure data for epidemiological studies (Gulia et al., 2015; Lin et al., 2017). Satellite data has been used to estimate ground pollution levels, either indirectly through proxies like Aerosol Optical Depth(AOD) for PM_2.5_ and PM_10_ or directly by using tropospheric NO_2_ measurements (de Hoogh et al., 2019; Stafoggia et al., 2017). Despite their ubiquity, satellite data alone cannot accurately capture ground-level concentrations and are affected by large measurement gaps due to cloud cover and sun-earth surface reflectance.

More recently, machine-learning (ML) methods have been used for exposure modelling, in many cases surpassing traditional techniques and providing more accurate estimates of pollutant exposure, especially at highly resolved spatial and temporal resolutions (Liu et al., 2022). ML approaches excel in utilizing diverse data sources, including land features, atmospheric variables, EDM outputs, and satellite data. By combining these sources with powerful and highly predictive algorithms, ML methods can offer a comprehensive reconstruction of pollution patterns over time and space. Various ML algorithms, such as random forest (Schneider et al., 2020; Stafoggia et al., 2020), neural networks (Di et al., 2016) and gradient boosting (Gutiérrez-Avila et al., 2022) have been used for this purpose. However, due to the recent adoption of ML in exposure modelling, choosing the right technique for a specific setting remains a significant challenge, with limited guidance available (Rybarczyk and Zalakeviciute, 2018). Given the wealth of ML algorithms available, recent developments in ML application to exposure assessment have focused on stacking ensemble methods (Breiman, 1996; van der Laan et al., 2007) (also referred to as Super Learner), combining outputs from different learners to further improve predictive accuracy and reduce over-fitting (Di et al., 2020; Shtein et al., 2020).

In this study, we aim to apply advanced ML-ensemble methods to reconstruct daily concentrations of NO_2_, PM_10_, and PM_2.5_ across Great Britain within the period 2003–2021 over a grid of 1 × 1km resolution.

## 2 Data

Our analysis relied on a Super Learner model trained with station measurements of the considered pollutants as the target, and a wealth of land-use, atmospheric and demographic data as predictors. This model was then used to predict the pollutant level across Great Britain.

This section summarises the geographical and temporal domain of the data, the pollution monitoring data, and the spatio-temporal and spatial predictors used in the analysis. A table with a list of data resources and links for each dataset is available in the appendix (Table S1).

### 2.1 Target geographical and temporal domain

Great Britain is the biggest of the British Isles and includes the countries of England, Scotland and Wales, with a total population of 67 million in 2021 (ONS, 2023a) and an area of 229,462 km^2^. According to the Köppen-Geigen classification, the climate is defined as temperate oceanic with warm summers and no dry seasons (Beck et al., 2018). The proposed reconstruction targeted 1-km square cells following the geometry of the British National Grid (OS, 2023a), resulting in a total of 242,851 grid cells. The daily reconstruction spanned the years 2003–2021.

### 2.2 Observations from ground monitoring stations

Ground observations were retrieved from five UK monitoring networks: Automatic Urban and Rural Network (AURN), Air Quality England (AQE), King’s College London (KCL), Scotland Air Quality Network (SAQN), and Wales Air Quality Network (WAQN) (DEFRA, 2024; ICL, 2024; RE&E, 2024; SG, 2024; WG, 2024). These measurements were complemented with data from two European Environmental Agency repositories, AirBase (EEA, 2014) and Air Quality-e-Reporting (EEA, 2024). The raw hourly observations, in micrograms per cubic metre (μg/m^3^), were processed by removing outlier measurements and discarding days with less than 75% completion (less than 18 hourly measures a day) to obtain representative daily averages. Additionally, we removed monitors with less than nine measurements per month and monitors with less than 270 recorded days per year. Finally, monitors from different sources placed at the same locations and displaying identical co-occurring observations were filtered-out, each time keeping the monitor with most observations overall. In the most common case of a full identical match, the AURN monitor was kept. Monitors were classified as either hotspot (traffic and industrial) or background (rural and suburban). Throughout the study period, 818 monitors provided 2, 874,994 daily observations for NO_2_, 1,737,318 for PM_10_, and 597,549 for PM_2.5_ (Table S2). Most monitors were clustered near cities and 682 had measurements of both NO_2_ and either PM.

### 2.3 Spatio-temporal predictors

Meteorological data was obtained from the ERA5 and ERA5-Land reanalysis datasets, with respective spatial resolutions of approximately 30 km and 9 km. Each predictor was sampled at two time-points (00:00 and 12:00) and all variables apart from boundary layer height were converted to a single daily average (Hersbach et al., 2020; Muñoz-Sabater et al., 2021). Relative humidity, wind speed, and wind direction were derived from dewpoint temperature and pressure data, and from wind-components, respectively. Formulas used to derive relative humidity and wind predictors can be found in Section S1 of the supplementary materials.

Total atmospheric column modelled AOD at 0.47, 0.67, 0.86, and 1.24 μm wavelengths as well as tropospheric NO_2_ were obtained from the EAC4-CAMS reanalysis database via the Copernicus Atmosphere Monitoring Service (CAMS) (Inness et al., 2019) at a sub-daily (00:00 and 12:00) and 80-km resolution. These were used to gap-fill the corresponding satellite derived products MCD19A2 and L3-OMNO2d which provide values of AOD and tropospheric cloud-screened NO_2_ at daily level and with a 1 km and 0.25° × 0.25° spatial resolutions, respectively (Krotkov et al., 2013; Lyapustin and Wang, 2018). As L3-OMNO2d data was only available from October 2004, the EAC4-CAMS NO_2_ column data was used in its place for the 2003 and 2004 models. Satellite data were obtained from two of NASA’s earth observation data services; the Land-Processes Distributed Active Archive Systems, and the Goddard Earth Sciences Data and Information System Centre (NASA, 2021a, 2021b, 2021c).

The European Modelling and Emissions Programme chemical transport model for the UK (EMEP4UK) was the source for modelled daily concentrations of PM_2.5_, PM_10_, NO_2_, nitrogen oxide (NO), sulphate (SO4), sulphur dioxide (SO2), dust, and sea salt at a 3 km spatial resolution (Scheffler and Vieno, 2022). Additionally, we used the 1-km resolution gridded annual output of PM_10_, PM_2.5_ and NO_2_ from the EDMs of the Department for Environment, Food & Rural Affairs (DEFRA, 2023).

The monthly and 1 km resolution normalized density vegetation index (NDVI) data product MOD13A3v6.1 was retrieved from NASA’s data services (Didan, 2021). Multi-year imperviousness data at 100m resolution was obtained from the Copernicus land monitoring service system (CLMS) library (CLMS, 2020).

### 2.4 Spatial predictors

Elevation data from the EU-DEM and land-cover type (100m) from the Corine Land Cover inventory were sourced from the CLMS (CLMS, 2016, 2019). Elevation was obtained at 25m resolution and was resampled to 1 km. We computed cell-by-cell standard deviation of the elevation to use it as a distinct predictor. Land cover data was processed, collapsing 44 distinct land classes into nine (Table S3), as in previous work (Schneider et al., 2020). We processed the resulting data into nine rasters, in which each cell value corresponded to the cell-fraction covered by that raster’s land class. Night-light data was retrieved as a 2015 annual composite from the VIIRS instrument onboard the SUOMI-NPP satellite, providing average radiance values at a spatial resolution of 750m (Elvidge et al., 2017).

A population density raster at a 1 km resolution based on the 2011 UK census was obtained from the Environment Information Data Centre (EIDC) (Reis et al., 2017). Road segment data was obtained from the “OpenRoads” initiative as a vector shapefile (OS, 2023b). Road density was calculated as the summed length of each road type (highway, secondary, local) within each 1-km cell. The annual average of daily traffic flow for all motor vehicles was obtained from the Department for Transport (DfT, 2023). We applied an ad-hoc linkage framework to reconstruct traffic flow data on highway and secondary roads (Section S2 of the supplementary materials).

Distance from each grid cell centroid to the sea, and to the ten major airports by passenger count was computed with geographical boundary data and airport locations sourced from the Civil Aviation Authority for the years 2015–2019 (CAA, 2023).

### 2.5 Other predictors

Time and location information of each measurement were used as predictors, including month, day of the week, day of the year, weekend indicators, and projected coordinates.

Six additional spatial predictors based on monitor measures were computed as detailed in previous work (Schneider et al., 2020). Four predictors represented leave-one-out inverse-distance weighted surfaces of annual averages computed independently for hotspot and background monitors, with power weights of 1 and 2. The other two variables represented the distance from each point to the nearest hotspot and background monitor.

To account for the coronavirus pandemic during the years 2020 and 2021, we added the aggregated daily number of lab-confirmed cases by country (England, Wales, Scotland) (ONS, 2023b), and the Stringency Index, a measure representing the severity of lockdown implementations at the same spatio-temporal scale (Hale et al., 2021). All predictors were harmonized prior to analysis as outlined in Section S3 (including Tables S10 and S11) of the supplementary materials.

## 3 Methods

The analysis was conducted in different stages, each time using a separate ML algorithm including shrinkage regression procedures, or their combination in an ensemble model. Specifically, the selected ML algorithms were random forest (RF) (Breiman, 2001), extreme gradient boosting (XGB) (Chen and Guestrin, 2016), light gradient boosting machine (LGBM) (Ke et al., 2017), while lasso and ridge were selected as linear but time-efficient regression alternatives (Hoerl and Kennard, 1970; Tibshirani, 1996). The various stages are described below.

### 3.1 Stage 1: reconstruction of PM_2.5_ at the monitoring sites

Daily observations and monitors providing PM_2.5_ data were very sparse compared to PM_10_. In the period 2003–2008, an average of 77,548 observations of PM_10_ were reported from 230 monitors each year in contrast with 5175 observations from 119 monitors for PM_2.5_ (Table S2). We therefore augmented the PM_2.5_ data by reconstructing concentrations in the series for monitors with co-located PM_10_ values, accounting for temporal indices (*day of week/year, month of year, and weekend indicator*), spatial coordinates (*easting/northing*) and monitor type (*hotspot/background)*. We chose to train an LGBM(Ke et al., 2017) model due to its increased efficiency and performance in internal trials against other methods and in previous research (Liu et al., 2023). We obtained optimal tuning parameters for each yearly model from a random grid search of 100 parameter combinations (Table S4). The model is represented by:

$$PM_{2.5(m,t)}^{y}=f(PM_{10(m,t)},\;type_{m},\;yday_{t},\;dow_{t},\;month_{t},\;weekend_{t},\;x_{m},\;y_{m})$$

### 3.2 Stage 2: reconstruction of MCD19A2 (AOD) and L3-OMNO2d (NO_2_) satellite observations

Ground PM and NO_2_ concentrations may be estimated from AOD and tropospheric column measurements of NO_2_ obtained from satellite data products (He et al., 2023; Stafoggia et al., 2019). As missingness is a pervasive issue with satellite data, reconstruction of these measurements has become common practice in data-driven air pollution modelling studies (Goldberg et al., 2019; Shtein et al., 2020).

We carried out yearly reconstructions of the daily product representing AOD at wavelengths of 0.47 and 0.55 μm (MCD19A2), and the product representing tropospheric cloud-screened columnar NO_2_ (L3-OMNO2d). In both cases, we used an RF algorithm, specified as follows:

$$\begin{array}{ll} Satellite.AOD_{i,t}^{\left(z.y\right)}= & f(CAMS.AOD_{i,t,0.47},CAMS.AOD_{i,t,0.55},CAMS.AOD_{i,t,0.67}\\ & ,CAMS.AOD_{i,t,0.865}CAMS^{2}.AOD_{i,t,1.24},yday_{t},{\text{x}}_{i},y_{i})\\ Satellite.AOD_{i,t}^{\left(z.y\right)}= & f(CAMS.AOD_{i,t,0.47},CAMS.AOD_{i,t,0.55},CAMS.AOD_{i,t,0.67}\\ & ,CAMS.AOD_{i,t,0.865}CAMS^{2}.AOD_{i,t,1.24},yday_{t},{\text{x}}_{i},y_{i}) \end{array}$$

*Satellite.AOD* at time *t* and wavelengths 0.47 and 0.55 μm (*z)* were modelled using the CAMS reanalysis data for AOD (*CAMS.AOD*) at the five wavelengths (0.47, 0.55, 0.67, 0.865 and 1.24 μm). The predictors for the *Satellite.NO*_*2*_ model were resampled to the resolution of the satellite data. These were mean daily boundary layer height (*BLH*), mean sea level pressure (*MSLP*), temperature (*T2M*), major roads density (*ROADS*), elevation (*ELEV*) and the percentage of urban and vegetation land-cover (*URBAN, VEGET*). With *i* indicating a grid cell and *t* indicating the Julian calendar day. Additionally, we included day of the year (*yday*) and grid cell centroid coordinates (*x,y*) as predictors in both models. Hyperparameter specification can be found in Section S4 of the supplementary materials.

### 3.3 Stage 3: ensemble spatio-temporal ML model of NO_2_, PM_10_, and PM_2.5_

We used a Super Learner method to estimate NO_2_, PM_10_, and PM_2.5_ in separate yearly models. Yearly models are fitted for two reasons: i) using the full range of predictors available each year given the difference in availability in earlier years; ii) computational convenience given the sheer amount of data considered. Super-learner is an ensemble method in which a meta-learner is used to optimally combine the predictions made by multiple candidate or base-learners (van der Laan et al., 2007).

We selected the RF, XGB, and LGBM algorithms as flexible base learners (Breiman, 2001; Chen and Guestrin, 2016; Ke et al., 2017, p. 20). The selection was based on practical reasons, whereby other learners such as neural networks could not be applied due to the high computational and time demand. Briefly, RF builds a large number of independent decision trees and computes its output as the averaged predictions from all trees. XGB and LGBM are both gradient-boosting algorithms that can be configured to operate with decision trees. Unlike RF, they construct the decision trees sequentially, aiming to improve their performance at each iteration. The difference between XGB and LGBM lies in the way the data to be fit is split within each tree. Technical details are provided in the references cited above. Additionally, we selected alternative regularized linear learners, lasso and ridge, for their computational efficiency (Hoerl and Kennard, 1970; Tibshirani, 1996).

A generalized additive model (GAM) was initially chosen as the meta-learner, following previous studies (Di et al., 2020; Shtein et al., 2020). GAMs estimate a response variable as smooth functions of predictors, and in previous applications allowed assigning spatially varying weights to different base-learners. However, we found that the GAM models made incongruent and extreme predictions at a later stage, due to the sparse and unbalanced monitor locations and the substantial spatial extrapolation involved. For this reason, we opted for a less flexible but more robust and established non-negative least squares model (NNLS), which has also been used as a meta-learner in air pollution mapping studies (Kianian et al., 2021; Yu et al., 2022, 2023). The NNLS algorithm is defined by the following formula:

$$\hat{f}(x)=\overset{m}{\underset{i=1}{\sum }}w_{i}f_{i}(x),w_{i}\geq 0$$

Differently from GAM, NNLS calculates the optimal coefficient or weighting (*w*_*i*_) for each base learner (*m*) from their predictions (*f*_*i*_*(x)*) restricting the weight values to positive values and constant across the domain, thus reducing artefacts due to spatial extrapolation.

Both LGBM and XGB algorithms were fine-tuned for each yearly model, selecting the best configuration from a random search of 100 hyperparameter combinations. To avoid the computationally demanding fine-tuning of the RF algorithm we used sensible and conventional defaults for the regression case (*500 trees, a third of randomly selected variables by split, and a minimum of 5 observations in each node*) (Probst et al., 2019). The predictors used in each yearly model were the same for the PM_2.5_ and PM_10_ models but not for the NO_2_ models (Table S5).

### 3.4 Performance assessment

We assessed the performance of all models by computing the coefficient of determination (R^2^), root-mean-square-error (RMSE), and intercept and slope values. These were obtained by fitting a simple linear model between observations and cross-validated predictions. We disambiguated the model performance statistics into three metrics representing spatial, temporal and overall performance, as described in previous work (Schneider et al., 2020).

For the reconstruction of PM_2.5_ and gap-filling of satellite data in stages 1 and 2, the prediction errors were respectively obtained via a monitor and cell-blocked 10-fold cross-validation procedure. After splitting the data into 10 groups, each including the entire set of observations from a given monitor or cell, the model was trained on 9-folds and used to predict on the 10th. After 10 iterations, the set of cross-validated predictions was used to compute the performance metrics.

To obtain cross-validated base and Super Learner predictions in stage 3, we also used a 10-fold monitor-blocked cross-validation strategy. This process, outlined in Section S5 of the supplementary materials, minimizes information leakage, and reproduces the task of predicting in locations not covered by monitors.

### 3.5 Stage 4: prediction of NO_2_, PM_10_ and PM_2.5_ over the full spatiotemporal domain

To generate the final predictions, we first trained the base-learners on the full observed data at monitoring sites, and then obtained out-of-sample predictions from each of them to fit the meta-learner. Finally, we used the fully fitted base and meta-learner models to predict over the entire study grid.

Variable importance measures were calculated from the fully fitted models, averaged across years, and standardized to obtain proportional contribution values. The contribution of each base learner to the ensemble was investigated by extracting the coefficients assigned to each input variable of the fitted NNLS models.

### 3.6 Software and R packages

All data processing and analysis were performed using the R statistical software on the RStudio integrated development environment (Posit Team, 2023; R Core Team, 2023). Monitoring data was downloaded via the *openair* (UK datasets) (Carslaw and Ropkins, 2012) and *saqgetr* (EU datasets) (Grange, 2019) packages. The ensemble ML framework was implemented using the *mlr3* package (Lang et al., 2019). The algorithmic engines used for the base learners were from the *ranger, xgboost, lightgbm* and *glmnet* R packages (Chen et al., 2023; Friedman et al., 2010; Shi et al., 2023; Wright and Ziegler, 2017).

## 4 Results

### 4.1 Stage 1 and 2: PM_2.5_ and satellite data reconstruction

The reconstruction of PM_2.5_ at monitoring sites in Stage 1 was carried out with yearly LGBM models. The R^2^ value ranged from 0.621 in 2006 to 0.926 in 2016 with an average of 0.801. Performance was lower in the spatial (R^2^ = 0.619) than in the temporal (R^2^ = 0.852) domain. Yearly detailed performance results can be found in the supplementary materials (Table S6).

The satellite data reconstruction through RF models in Stage 2 displayed very good performance overall. The NO_2_ reconstruction models were the worst performing with R^2^ = 0.807. The products measuring AOD wavelengths at 0.47 and 0.55 μm were reconstructed at a higher accuracy (R^2^ = 0.958) (Table S7).

### 4.2 Stage 3: ensemble spatio-temporal ML model of NO_2_, PM_10_, and PM_2.5_

The main results of Stage 3 are shown in Table 1 as three period averages representing early (2003–2008), middle (2009–2014) and recent (2015–2021) years which correspond to generally higher, middling, and lower countrywide air pollution levels (Fig. S5). The total average performance of the NNLS-ensemble given by R^2^ was good for the NO_2_ (R^2^ = 0.690) and the PM_10_ (R^2^ = 0.704) models, and very good in the case of the PM_2.5_ models (R^2^ = 0.820). As expected, the ensemble performance was improved, albeit minimally, relative to all base learners, and in some years the strongest base learner outperformed the ensemble (Fig. S1). It is worth noting that the three tree-based base learners (RF, XGB, and LGBM) show high and similar accuracy, while the two alternative learners based on regularized regression (ridge and lasso) have a much lower performance, possibly due to their strong functional assumptions (linear relationships and lack of interactions) (Table 1). Performance was highest across all pollutants and models in the most recent period (2015–2021), presumably due to higher availability of pollutant measurements and/or improved predictor accuracy (performance statistics by year are shown in Fig. S2). All pollutant models display a consistent and marked decrease in RMSE in the later years, although with only a slight increase in R^2^, probably due to the narrower concentration range in the training data owing to falling polluting levels across the study period.

Focussing on the 15 most predictive features on average across all years (Table S8), we found day of the year and spatial lag variables to be the most represented. Distance to nearest monitor was more important in NO_2_ than PM models, where the annual inverse-distance-weighted features were more predictive. Residential and work population density were highly predictive although only for NO_2_ models. The most important predictors across all pollutants and ML algorithms were pollutant-specific outputs from the EMEP4UK model, followed by atmospheric reanalysis features and spatial lag variables, in particular precipitation and wind direction for the PM models and temperature and total column NO_2_ for the NO_2_ models.

While the ensemble model improved on the predictive performance of the base learners in the temporal dimension, the same was not true for the spatial aspect (Table S9). Averaged across all years, the spatial performance of the NNLS model was lower than that of the strongest base-learner. This difference was very small but persistent across the entire study period (Fig. S1) and can be due to the constraint of the NNLS meta-learners to produce spatially constant weights for the base learners.

The fitted NNLS models, which act as meta-learners, can be examined to identify which learner was considered to contribute most and least to the ensemble model. In Fig. S3, we show that weak learner predictions were rarely, if at all considered, while XGB predictions were consistently the maximum contributors. Fig. S4 shows the comparison of observations and cross-validated predictions for the year 2019. As expected, the LASSO and RIDGE learner predictions exhibit the weakest correlation to the observed values as well as some degree of bias, while all NO_2_ model predictions appear more distant from the bisector line due to their higher error and lower R^2^. The meta-learner (NNLS) panels exemplify the very strong performance of the ensemble model for the three pollutants. In particular, we note its unbiasedness as indicated by the intercept and slope of the fitted regression line.

### 4.3 Prediction of NO_2_ and PM_2.5_ over the full spatio-temporal domain

Daily mapping of a single pollutant for one year over the British National Grid yielded 88,640,615 cell-values. Fig. S5 shows the average trend for each pollutant over the study period. An overall decreasing pattern can be observed. However, the decrease is not uniform, with the strongest reduction occurring in the late 2000s.

**Fig. 1:**
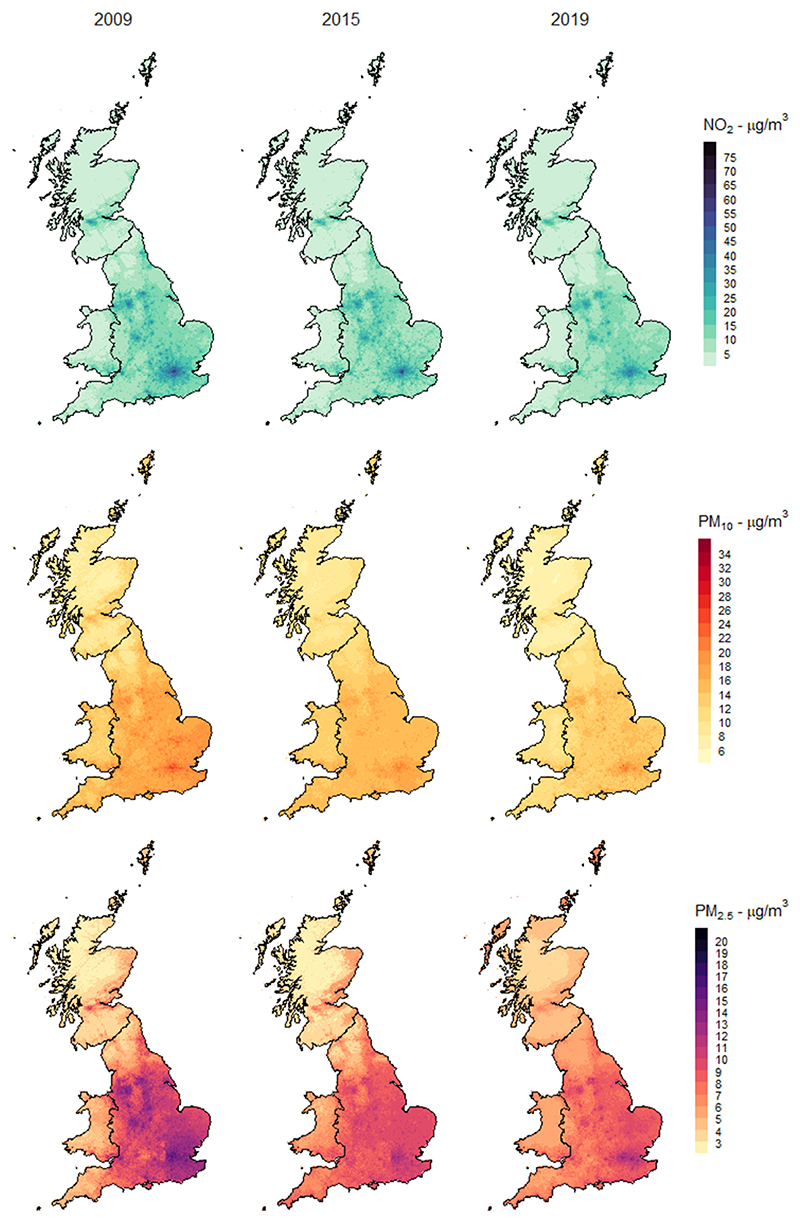
Annual average maps. Levels in micrograms per cubic metre of NO_2_, PM_10_, and PM_2.5_ in the years 2009, 2015, and 2019. Note each map uses a different range and colour-value correspondence to magnify spatial and temporal differences for each pollutant.

This decreasing trend is also captured in Fig. 1, which shows annual average maps for each pollutant for the years 2009, 2015 and 2019. As expected, all three pollutants show strong spatial heterogeneity at the national scale, with higher concentrations found in urban agglomerations and areas of higher traffic intensity. In contrast to PM, NO_2_ displays spatial heterogeneity at a smaller scale, with peaks in urban areas and high-traffic roads. In the NO_2_ maps, we can recognise traffic arteries as they cut through rural areas and create a high contrast gradient in NO_2_ levels. Differently, PM_10_ and PM_2.5_ are characterized by large-scale variations and regional differences.

**Fig. 2:**
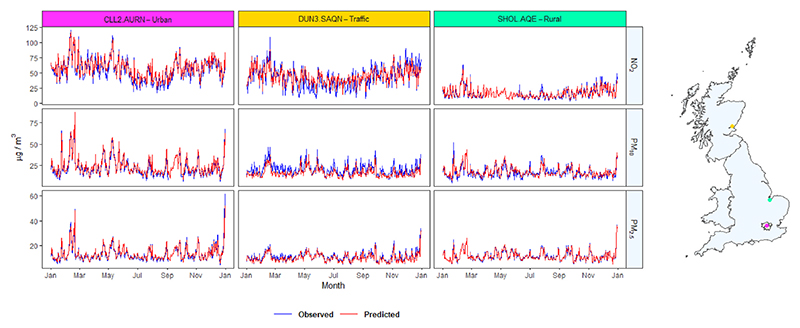
Time series of observed and predicted NO_2_, PM_10_, and PM_2.5_ throughout 2018 for three specific monitors of different types. Levels are in micrograms per cubic metre and shown at three different monitoring sites with the corresponding grid-cell locations in Great Britain.

Fig. 2 shows nine daily time series comparing observation data at three monitoring stations with the predicted concentrations in their respective grid-cell, and their location within Great Britain. The graphs indicate that the ensemble model predicts with high accuracy in the temporal dimension, and this is visually apparent from how closely predictions trace observations across an entire year. In this sample, the NO_2_ and PM_10_ models show a lower performance at the traffic monitor. This is likely due to the difficulty of models fitted using 1-km gridded data to capture local differences in high-traffic areas, especially for NO_2_ which is characterised by higher spatial variation. Nonetheless, the prediction is still fairly good, especially in capturing temporal differences.

**Fig. 3:**
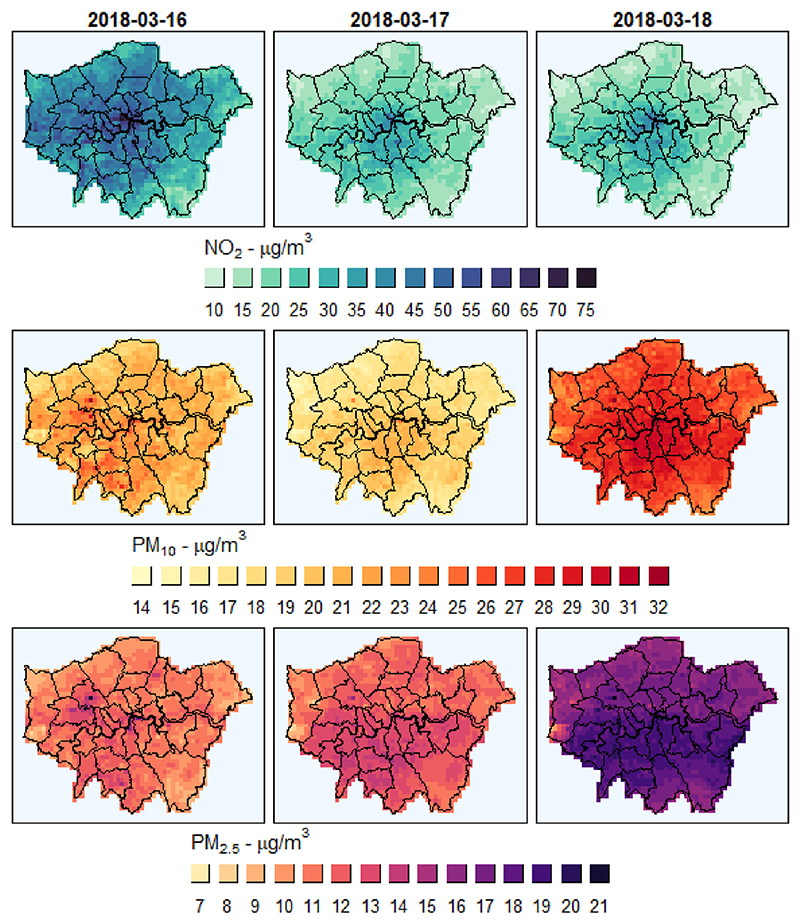
Daily predicted concentrations of NO_2_, PM_10_, and PM_2.5_ over the region of Greater London. Levels are in micrograms per cubic metre and span from Friday 16th to Sunday 18th of March of 2018. Each map uses a different range and colour-value correspondence to magnify spatial and temporal differences for each pollutant.

In Fig. 3, we show the degree of spatiotemporal variation predicted at a sub-national scale by mapping pollutant levels for three consecutive days starting from Friday 16th of March over the smallest region of Great Britain. Temporal changes in pollutant levels are clearly visible, with some being tied to human activity such as the elevated NO_2_ levels decreasing during the week-end due to the change in traffic. Different spatial heterogeneity for the two pollutant types can be appreciated at this scale, with PM showing larger differences across wider areas, (the subtle north-south difference on 2019-03-18), and NO_2_ displaying much more localised and spatially stable concentration peaks.

## 5 Discussion and conclusion

This study combines state-of-the-art ML techniques with a comprehensive environmental feature dataset to map daily levels of air pollutants in Great Britain from 2003 to 2021. Model performance across all years is good for PM_10_ and NO_2_ and excellent for PM_2.5_, with better performance observed in the later periods and in the temporal dimension. The output generated from the ML ensemble models consists of 5 billion data-points corresponding to 1kmx1km and daily resolved air pollution values, and it is unique in its combination of spatio-temporal resolution and coverage in the UK. This data may contribute substantially to epidemiological research on the short- and long-term health effects of air pollution as daily or annual-averaged exposure.

Three recent studies produced similar data on the same geographic area and a subset of the period modelled here. Liu and colleagues (Liu et al., 2023) trained a fine-tuned LGBM algorithm to predict daily and 1 km resolved PM_2.5_ levels from 1980 to 2019. The model’s performance for daily prediction in the common years 2010–2019, as measured by an R^2^ of 0.72, is lower than the present results. However, the model developed is trained on multi-year data, employs a single powerful learner for increased efficiency, and focuses on back-extrapolation, which complicates the comparison with the present single-year models. Wang and colleagues (Wang et al., 2022) developed very high spatial resolution models for daily predictions of four pollutants in the 2011–2015 period. The models are underpinned by a two-stage GAM dealing with time-varying and time-invariant dimensions separately and produced predictions at far higher resolution (25 m). However, the performances by R^2^ reported for the daily NO_2_, PM_10_ and PM_2.5_ models are 0.63, 0.80, and 0.77, respectively, similar to those achieved at 1-km resolution with our ensemble models in comparable years. A closer comparison is possible with our previous work (Schneider et al., 2020), where we used a simpler modelling framework which we developed further with data and methodological improvements. Specifically, we integrated an increased number of ground monitor observations, updated environmental datasets, and applied an advanced ensemble machine-learning method while extending temporal coverage by eight years, as well as adding NO_2_ and PM_10_ to the pollutant set. As a result, we achieve an improvement in performance in PM_2.5_ prediction, from an average R^2^ of 0.77–0.83 for the common years 2008–2018 (Schneider et al., 2020).

Within the ensemble framework, the stronger ML-based learners exhibited similarly high and consistent performance (Fig. S2). For all the base learners, satellite products like AOD and TCNO2 were considered, but their importance was limited, in agreement with similar studies (Tian et al., 2023; Yu et al., 2022). Despite their low predictive importance, we decided not to exclude them from the predictor set as they provide data for sparsely monitored areas.

The study setting included an unequal temporal and geographical distribution of ground monitor observations. Specifically, many more observations were available in the second half of the study period, and ground monitors show a clear spatial clustering in urban areas and low coverage in remote regions (Fig. S6). We initially explored the use of a GAM meta-learner, consistently with previous studies (Dimakopoulou et al., 2022; He et al., 2023), but we found that the spline-based extrapolation of the prediction in sparsely covered areas resulted in unrealistically extreme values, especially at the boundaries. We advise careful consideration when using such flexible methods. While the NNLS regression model we ultimately chose for our ensemble modelling showed very good performance, efficiency, and no abnormal predictions, it has the limitation that it cannot assign spatially-varying weights to the different base learners. We encourage future research to fill this gap in ML for exposure modelling. We validated our ensemble with ten-fold cross-validation allowing for nearby observations to inform each model. This strategy is a distant variant of spatial blocking, where large areas are defined as cross-validation folds, and which has been deemed to return overly pessimistic performance estimates (Wadoux et al., 2021).

This study faces three primary limitations. First, the clustered distribution of monitoring stations, predominantly found in urban areas, may introduce biases in the training dataset, limiting the generalizability of our models to rural regions. Extrapolation to areas with low data availability due to sub-optimal spatial sampling is a central issue in predictive mapping studies (Meyer and Pebesma, 2021; Wadoux et al., 2021). Second, resolution-related challenges arise from transforming and harmonizing covariates to different spatial resolutions, which impacts the characterization of spatial variability. Furthermore, while the 1 km resolution suits epidemiological studies, it may not fully capture the nuances in pollutant concentrations, especially in urban areas (Tadić et al., 2015) or for rapidly decaying and spatially heterogeneous gaseous pollutants like NO_2_. Third, our use of ensemble learning with a restricted few powerful algorithms diverges from the original recommendation of combining numerous, if less flexible learners (van der Laan et al., 2007). However, our approach is the most commonly applied in the literature (Danesh Yazdi et al., 2020; He et al., 2023) and, in this analysis, resulted in a very small improvement of the ensemble over the base learners.

To conclude, we present two potential research directions that would benefit the advancement of data-driven air pollution modelling. The first involves further developing of model and map validation strategies, such as sampling-intensity weighted cross-validation (de Bruin et al., 2022) and k-fold nearest neighbour distance matching-CV (Linnenbrink et al., 2023). These approaches might facilitate performance comparison between distinct models and provide more accurate estimates of model performance. Second, we underscore the need for high performance and very high-resolution models, aimed at providing pollutant exposure predictions at point locations instead of within cells of a pre-determined grid. This would enable increasingly accurate exposure predictions and assessment, benefitting epidemiological research and public health by an improved ability to estimate health risks attributable to environmental factors.

This study employed cutting-edge spatial data science and machine learning methods to map air pollution across Great Britain from 2003 to 2021, achieving notable accuracy across all pollutants. Building upon and enhancing an existing approach, we integrated a larger set of data and an improved machine learning framework, leading to a significant improvement in PM_2.5_ prediction as well as new data on PM_10_ and NO_2_. With a high spatiotemporal resolution and performance, this framework has the potential to generate reliable exposure values over a large geographic and temporal domain and thus power a wide range of health and environmental research.

## Supplementary Material

Appendix

## Figures and Tables

**Table 1:** Three period-specific (2003–2008, 2009–2014, 2015–2021) means of overall R^2^, RMSE (μg/m^3^), slope (μg/m^3^), and intercept (μg/m^3^) for each base learner and the ensemble learner. Higher R^2^ and lower RMSE values indicate increased power to explain the trends in the observed data and smaller errors when comparing predictions and observations, respectively.

Stage 3 - Single and Ensemble learner overall cross-validated performance																									
		*RF*				*XGB*				*LGBM*				*RIDGE*				*LASSO*				*NNLS*			
		R^2^	RMSE	Inter.	Slope	R^2^	RMSE	Inter.	Slope	R^2^	RMSE	Inter.	Slope	R^2^	RMSE	Inter.	Slope	R^2^	RMSE	Inter.	Slope	R^2^	RMSE	Inter.	Slope
NO_2_	2003- 2008	0.693	13.246	1.142	1.032	0.704	12.994	2.054	1.007	0.697	13.153	1.850	1.003	0.498	16.965	6.340	0.901	0.491	17.074	6.976	0.878	0.705	12.983	1.764	1.004
	2009- 2014	0.647	14.225	2.025	1.018	0.651	14.144	2.531	1.002	0.643	14.298	2.656	0.990	0.499	16.956	6.306	0.904	0.498	16.970	6.626	0.888	0.653	14.103	2.302	1.000
	2015- 2021	0.704	10.228	0.649	1.052	0.708	10.153	1.128	1.033	0.694	10.386	1.337	1.014	0.522	12.934	4.478	0.920	0.518	12.991	4.920	0.896	0.712	10.080	0.920	1.023
	**Mean**	**0.681**	**12.566**	**1.272**	**1.034**	**0.688**	**12.430**	**1.904**	**1.014**	**0.678**	**12.612**	**1.947**	**1.002**	**0.507**	**15.618**	**5.708**	**0.908**	**0.503**	**15.678**	**6.174**	**0.887**	**0.690**	**12.389**	**1.662**	**1.009**
PM_10_	2003- 2008	0.670	7.799	0.027	1.038	0.677	7.724	0.624	1.014	0.667	7.842	0.548	1.013	0.448	10.113	-0.604	1.095	0.452	10.078	0.479	1.044	0.679	7.702	0.301	1.024
	2009- 2014	0.692	6.448	-0.112	1.051	0.700	6.372	0.331	1.028	0.690	6.466	0.430	1.019	0.461	8.541	-1.404	1.148	0.470	8.473	-0.699	1.107	0.700	6.372	0.153	1.033
	2015- 2021	0.727	5.108	-0.134	1.056	0.734	5.048	0.113	1.053	0.726	5.122	0.216	1.028	0.477	7.081	-1.037	1.148	0.483	7.042	-0.404	1.102	0.735	5.041	0.164	1.029
	**Mean**	**0.697**	**6.452**	**-0.073**	**1.048**	**0.704**	**6.381**	**0.356**	**1.032**	**0.694**	**6.477**	**0.398**	**1.020**	**0.462**	**8.578**	**-1.015**	**1.130**	**0468**	**8.531**	**-0.208**	**1.085**	**0.704**	**6.371**	**0.206**	**1.029**
PM_2.5_	2003- 2008	0.784	3.256	-0.091	1.037	0.787	3.238	0.263	1.007	0.782	3.267	0.124	1.016	0.551	4.699	-0.801	1.130	0.557	4.668	-0.261	1.077	0.789	3.220	0.090	1.020
	2009- 2014	0.807	3.658	-0.147	1.055	0.813	3.598	0.170	1.029	0.809	3.640	0.145	1.025	0.575	5.427	-0.869	1.146	0.580	5.392	-0.482	1.108	0.816	3.570	0.119	1.024
	2015- 2021	0.851	2.681	-0.033	1.048	0.852	2.670	0.149	1.024	0.848	2.705	0.102	1.028	0.591	4.457	-1.105	1.217	0.597	4.420	-0.685	1.160	0.856	2.635	0.107	1.022
	**Mean**	**0.814**	**3.198**	**-** **0.090**	**1.046**	**0.817**	**3.169**	**0.194**	**1.020**	**0.813**	**3.204**	**0.124**	**1.023**	**0.573**	**4.861**	**-0.925**	**1.164**	**0.578**	**4.827**	**-0.476**	**1.115**	**0.820**	**3.142**	**0.105**	**1.022**
