# Supplementary material for: High resolution mapping of nitrogen dioxide and particulate matter in Great Britain (2003–2021) with multi-stage data reconstruction and ensemble machine learning methods
Source: Atmos Pollut Res. Author manuscript; Available in PMC 2024 Nov 1. (PMC7616380; doi:10.1016/j.apr.2024.102284)
Supplement: Appendix [file EMS198008-supplement-Appendix.pdf]

Supplementary Material for

Title: High resolution mapping of nitrogen dioxide and particulate matter in Great Britain (2003-2021) with multi-stage data reconstruction and ensemble machine learning methods

Authors:
de la Cruz Libardi, Arturo [a] ; Arturo.de-la-Cruz-Libardi@lshtm.ac.uk;
Masselot, Pierre [a] ;
Schneider, Rochelle [ef];
Nightingale, Emily [c];
Milojevic, Ai [bd];
Vanoli, Jacopo [ag];
Mistry, Malcolm N.[ah];
Gasparrini, Antonio [a];

Affiliations:
[a] Environment & Health Modelling (EHM) Lab, Department of Public Health Environments and Society, London School of Hygiene & Tropical Medicine, 15-17 Tavistock Place, WC1H 9SH London, United Kingdom
[b] Department of Public Health, Environments and Society, London School of Hygiene & Tropical Medicine, 15-17 Tavistock Place, WC1H 9SH London, United Kingdom
[c] Department of Global Health and Development, London School of Hygiene & Tropical Medicine, Keppel Street, WC1E 7HT, London, United Kingdom
[d] Centre on Climate Change & Planetary Health, London School of Hygiene & Tropical Medicine, Keppel Street, WC1E 7HT, London, United Kingdom
[e] Φ-lab (Phi-lab), European Space Agency (ESA), Frascati, Italy
[f] Forecast Department, European Centre for Medium-Range Weather Forecast (ECMWF), Reading, United Kingdom
[g] School of Tropical Medicine and Global Health, Nagasaki University, Nagasaki, Japan
[h] Department of Economics, Ca' Foscari University of Venice, Italy

Corresponding author: Arturo de la Cruz Libardi, Arturo.de-la-Cruz-Libardi@lshtm.ac.uk; London School of Hygiene & Tropical Medicine, 15-17 Tavistock Place, WC1H 9SH London, United Kingdom

Table of Contents

Section 1: Relative humidity, wind direction and wind speed equations 3

Relative humidity (in %) computation using temperature and dewpoint temperature. 3

Wind direction in radians 3

Wind speed in m/s 3

Section 2: Traffic data linkage and reconstruction 4

Linkage of the Ordnance Survey Open Roads dataset with annual traffic-flow data 4

Reconstruction of road traffic flow annual averages 4

Rasterization of linked traffic data 4

Section 3: Data harmonisation 5

Section 4: Stage 2 hyperparameter specification 6

Section 5: Ensemble-model cross validation 7

Figure S1: Ensemble improvement over base-learners 8

Figure S2: Base- and meta-learner yearly performance 9

Figure S3: Base-learner contribution 10

Figure S4: Observed vs predicted density scatterplot 11

Figure S5: Annual average over entire study grid. 12

Figure S6: Map of contributing NO_2 and PM monitors in 2003 and 2021. 13

Table S1: Summary of datasets and products used. 14

Table S2: Observations (days), number of contributing monitors, and yearly mean (ug/m^3) of NO_2, PM_{10}, and $PM_{2.5}$. 17

Table S3: Land cover reclassification. 18

Table S4: Yearly hyperparameters for the Light Gradient Boosting Machine algorithm. 20

Table S5: Variables included in stage 3 base-learner models. 21

Table S6: Stage 1, overall, spatial, and temporal performance results of yearly LGBM models. 23

Table S7: Stage 2, overall model performance results for satellite data reconstruction. 24

Table S8: Top 15 features by average relative importance by ML algorithm and pollutant modelled. 26

Table S9: Total average spatial and temporal performance 27

Table S10: Summaries of predictor values at monitor locations. 28

Table S11: Summaries of variables over prediction grid 30

References 32

Section 1: Relative humidity, wind direction and wind speed equations

Relative humidity (in %) computation using temperature and dewpoint temperature.

R code and formula (eq. 1) for the calculation of relative humidity (%) from dewpoint temperature (*d2m*) and ambient temperature (*temp*) adapted from the National Physics Laboratory (National Physics Laboratory, 2023).

```
 rh <- ((6.11*exp(((2.456*10^6)/461)*((1/273)-(1/d2m)))) /
(6.11*exp(((2.456*10^6)/461)*((1/273)-(1/temp)))) )*100
```

$$\frac{6.11\times\exp\left(\frac{2.456\times10^{6}}{461}\times\left(\frac{1}{273}-\frac{1}{\mathrm{d2m}}\right)\right)}{6.11\times\exp\left(\frac{2.456\times10^{6}}{461}\times\left(\frac{1}{273}-\frac{1}{\mathrm{temp}}\right)\right)}\times 100 \qquad (1)$$

Wind direction in radians

R code and formulas used to obtain wind direction (eq. 2) and speed (eq. 3) from (*u, v*) wind components. Adapted from the Copernicus ERA5 database manual (Copernicus Knowledge Base, 2023).

```
wind_direction <- (270-atan2(v,u)*180/pi) %% 360
```

$$\mathit{Wind\ direction} = \left(270 - \frac{\mathrm{atan2}(v,u)\times 180}{\pi}\right)\backslash\mathrm{mod}360 \qquad (2)$$

Wind speed in m/s

```
wind_speed <- sqrt((u^2)+(v^2))
```

$$\mathit{Wind\ speed} = \sqrt{u^2 + v^2} \qquad (3)$$

Section 2: Traffic data linkage and reconstruction

Linkage of the Ordnance Survey Open Roads dataset with annual traffic-flow data

The OS OpenRoads dataset is a high level, link-and-node map of motorways, primary, secondary, and local roads in the UK (OS, 2023). The link features identify unique road segments, which have attributes describing their length, road properties and their location. The traffic dataset (DfT, 2023) consists of count-points, each identified by the name and category of the road they lie on, as well as by their coordinates, and unique id label. Finally, count-points are associated with a yearly traffic-flow value.

We considered count points to be linkable if they lied on a named road. Importantly, almost 19% of count points had “U”, “C”, and “B” as road names indicating their belonging to unclassified, and unnamed B and C-type roads. We first matched count-points with segments in the OS dataset, linking them by road name. Within this set, we paired each segment with the nearest count-point, and we repeated this for all named roads for which we have count-points.

Two facts are to be noted. First, 98% of the roads present in the traffic data were also found in the OS roads dataset. This meant that nearly all the linkable traffic count points were evaluated to be linked to road segments. Second, a single count-point may have been linked to several road segments, and similarly, a count point may have remained unlinked to any road segment. This would have occurred when a count-point was the nearest to many segments or when it was not the nearest to any segment. A manual check comparing the total linkable count points, and the eventual linked count points determined that nearly all (97%) count points were linked to road segments.

Reconstruction of road traffic flow annual averages

We used the linked dataset of segment-count-points to reconstruct annual traffic values for all major and secondary road segments in the OS roads dataset. First, we computed each road segment’s centroid, and used them to extract predictor values. Then, we computed the mean traffic values by segment id (ID.MEAN). Finally, we specified and trained a random forest model (eq. 4), which we used to predict missing yearly traffic values.

$$Annual.Traffic_i^{(y)} = f\begin{pmatrix} ID.MEAN_i, ROAD.CAT_i, ELEV_i, DIST.AIRP_i, LC.URBAN_i, \dots , LC.BARE_i, \\ NDVI_{i,y}, NIGHT.LIGHT_i, RES.POP_i, WORK.POP_i, x_i, y_i, year_y \end{pmatrix} \quad (4)$$

We set default hyperparameters *(mtry=5,num.trees=500,min.node.size=5)*. In the equation, *i* indicates the segment id and *y* refers to the year. The model performance was very high, with a 10-fold cross-validated R^2 of 0.96.

Rasterization of linked traffic data

We translated segment-level traffic data to grid-cell-level data. First, we intersected the segment geometries with the grid, resulting in many more, smaller, segments, each only spanning one grid cell at most. Then, we standardized the traffic values for each segment, considering their new length. Lastly, we summed each segment’s standardized values by cell, resulting in a gridded traffic dataset, with a traffic value per $1km^2$.

Section 3: Data harmonisation

Predictor values were calculated at both the monitor locations and over the entire study area (see Tables S10 and S11 in this document). Specifically, all predictors were harmonised by resampling their values to 1-km resolution, matching the underlying grid cell structure of the British National Grid. To achieve complete coverage of the study grid, a small number of predictors required minimal filling which was carried out by bilinear interpolation, nearest-cell value, or by 0-value filling in the case of population data. The spatial lag predictors were calculated as points corresponding to monitor locations for use in the modelling stage, and in grid format (using the cell centroids) for use in the prediction stage.

Section 4: Stage 2 hyperparameter specification

The RF model is defined with three key hyperparameters: *mtry* controls the number of randomly selected variables at each decision tree split, *min.node.size* refers to the minimum number of observations at a node that will cease the tree-fitting procedure, and *num. trees* indicates how many decision trees to fit as part of the random forest algorithm (Wright and Ziegler, 2017). In this case, the RF hyperparameters were chosen from previous work (Schneider et al., 2020) for the *Satellite.AOD* model *(50 trees, 4 randomly selected variables by split, and a minimum of 5 observations in each node)* and from default settings for the *Satellite.*NO_2 model *(500 trees, 2 randomly selected variables by split, and a minimum of 5 observations in each node).*

Section 5: Ensemble-model cross validation

These are the cross-validation steps taken to evaluate ensemble-model performance. The dataset refers to the input to the machine-learning modelling stage (Stage 3).

1. We divided our dataset into ten monitor-blocked folds, ensuring that all observations from the same monitor were contained within the same fold.
2. We obtained out-of-sample predictions for the entire dataset from each base learner (RF, XGB, LGBM, RIDGE, LASSO) by resampling on the partitioned dataset. Each learner was iteratively fit on nine folds and then used to predict the remaining fold.
3. Then we carried out the same process to obtain cross-validated predictions from the meta-learner algorithm using the predictions from Step 2. This cross-validation was also monitor-blocked as it employed the same fold partitioning set in Step 1.

We opted for the above method as it employed one-tenth of the computing time and showed almost identical performance results to the full-outer cross-validation of the ensemble, its steps are outlined below.

1. We divided our dataset into ten monitor-blocked folds, ensuring all observations from the same monitor were contained within a single fold. We repeated this grouping ten times, each time defining a test dataset represented by a single fold and a training dataset using the other nine folds.
2. At each iteration, we first fitted each base learner on the train dataset, and then obtained out-of-sample predictions for the train data using monitor-blocked cross-validation.
3. We used the out-of-sample predictions from the previous step to fit the meta-learner on the same train dataset.
4. Finally, we used each fitted base learner to predict on the test dataset, and then passed such output to the meta-leaner to obtain predictions on the same data.

We repeated steps 2-4 until we had obtained predictions for the entire dataset, from which we calculated performance statistics.

Figure S1: Ensemble improvement over base-learners

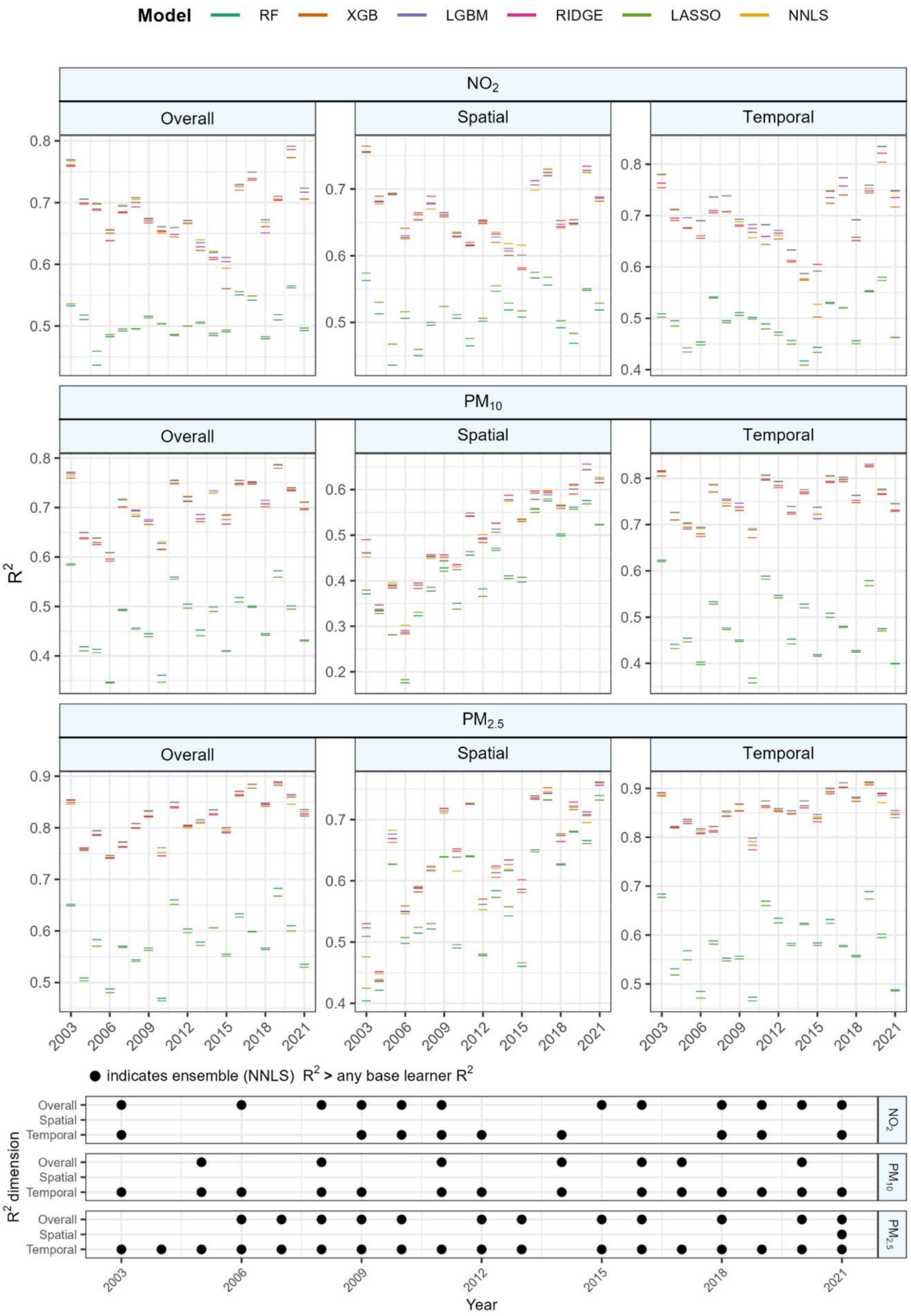

Figure 1: Comparison of R^2 values between base- and meta-learner. top) yearly R^2 value by pollutant and dimension. bottom) visualisation of whether any base-learner out-performed the meta-learner.

Figure S2: Base- and meta-learner yearly performance

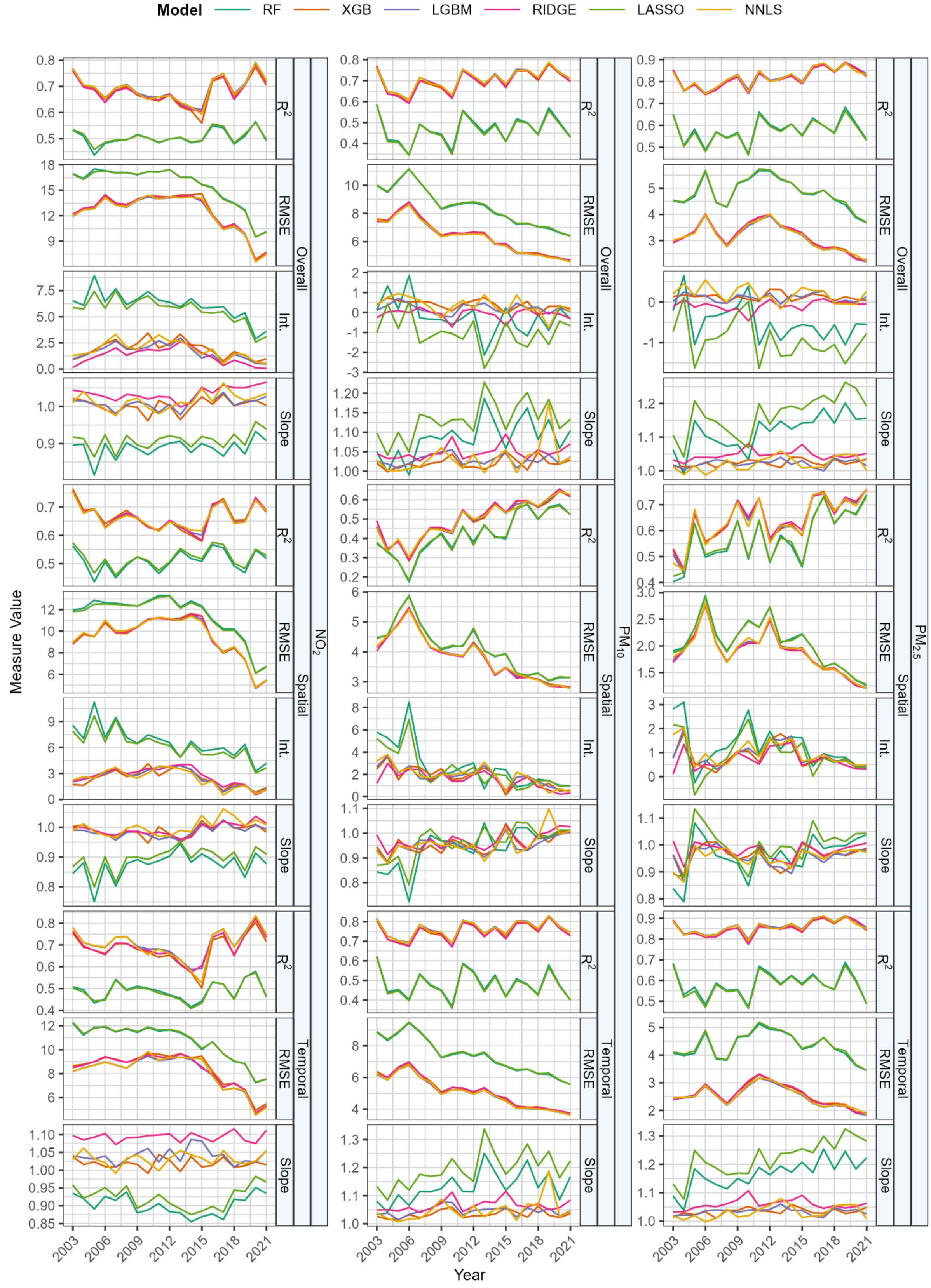

Figure 2: Yearly performance statistics computed. Intercept values of temporal dimension not shown as all ≈ 0. RMSE, Intercept and Slope units are ug/m^3

Figure S3: Base-learner contribution

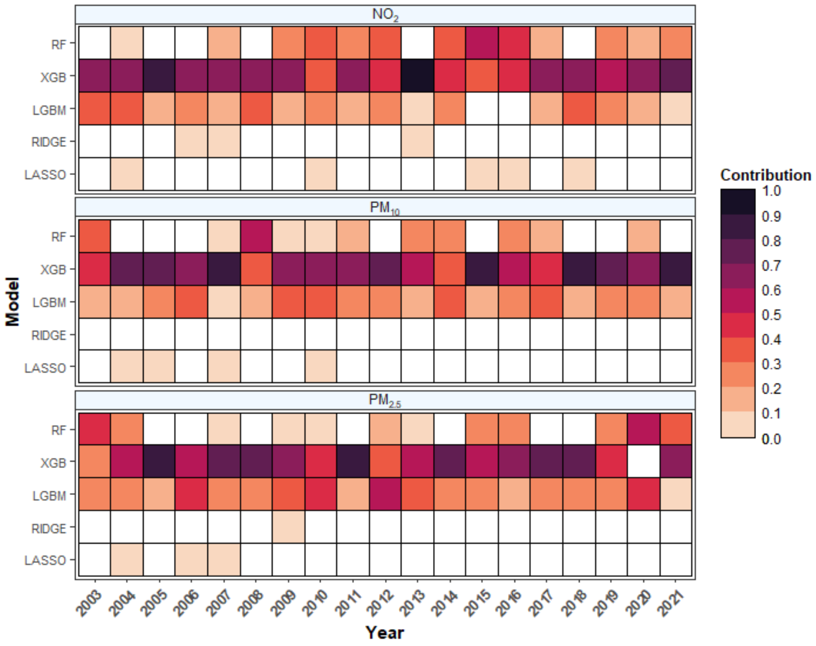

Figure 3: Base-learner contribution to the prediction of each pollutant (NO_2, $PM_{2.5}$, and PM_{10}) by year (2003-2021), represented as coefficients of the non-negative least squares meta-learner.

Figure S4: Observed vs predicted density scatterplot

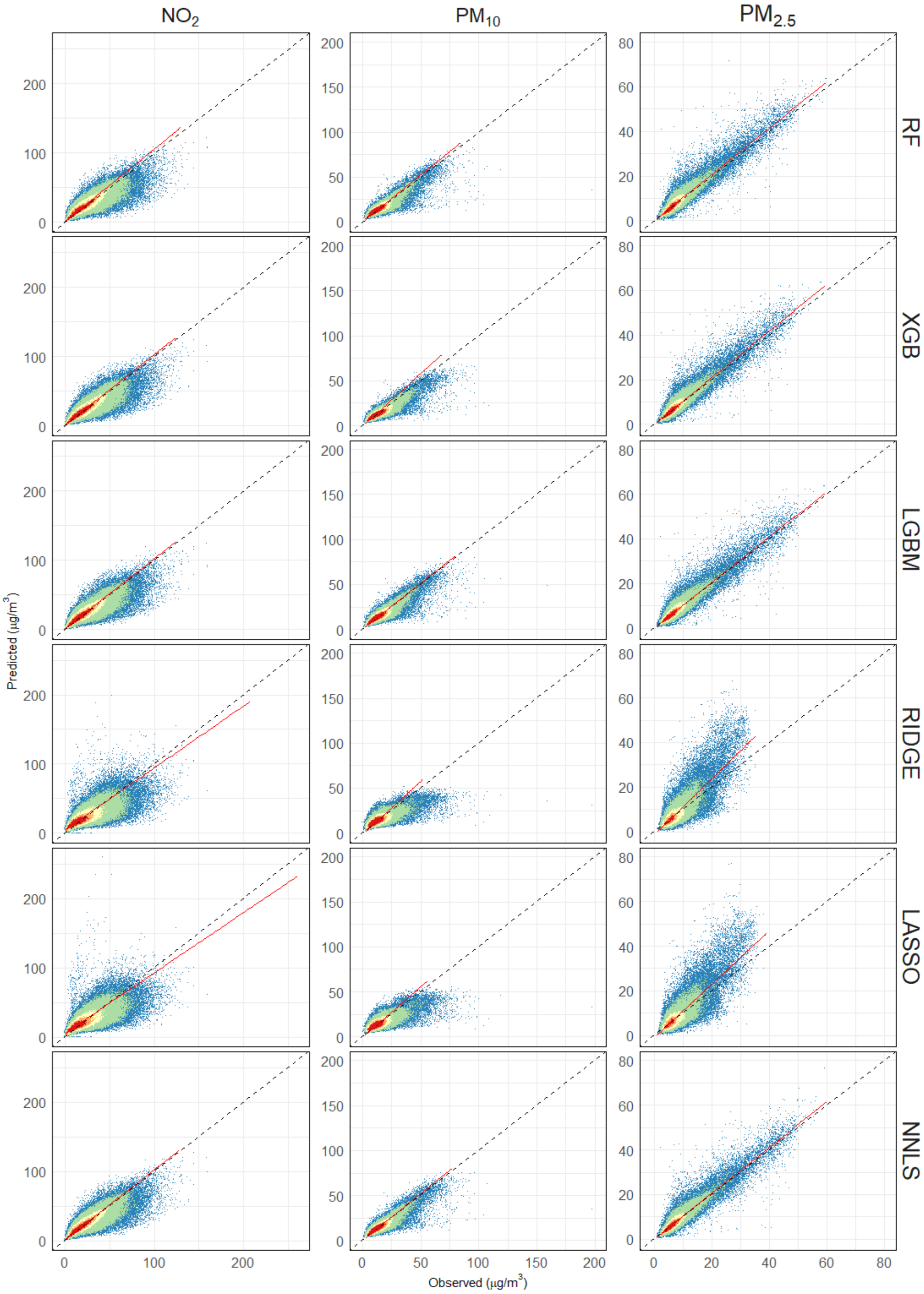

Figure 4: Hexagon-binned density and regression line of cross-validated predictions against observed values in 2019. All base-learners (RF, XGB, LGBM, RIDGE, LASSO), and the ensemble (NNLS) are shown for each pollutant (NO_2, PM_{10} and $PM_{2.5}$). Bisector in dashed black. Axis limits are symmetrical and fixed by pollutant for ease of comparison.

Figure S5: Annual average over entire study grid.

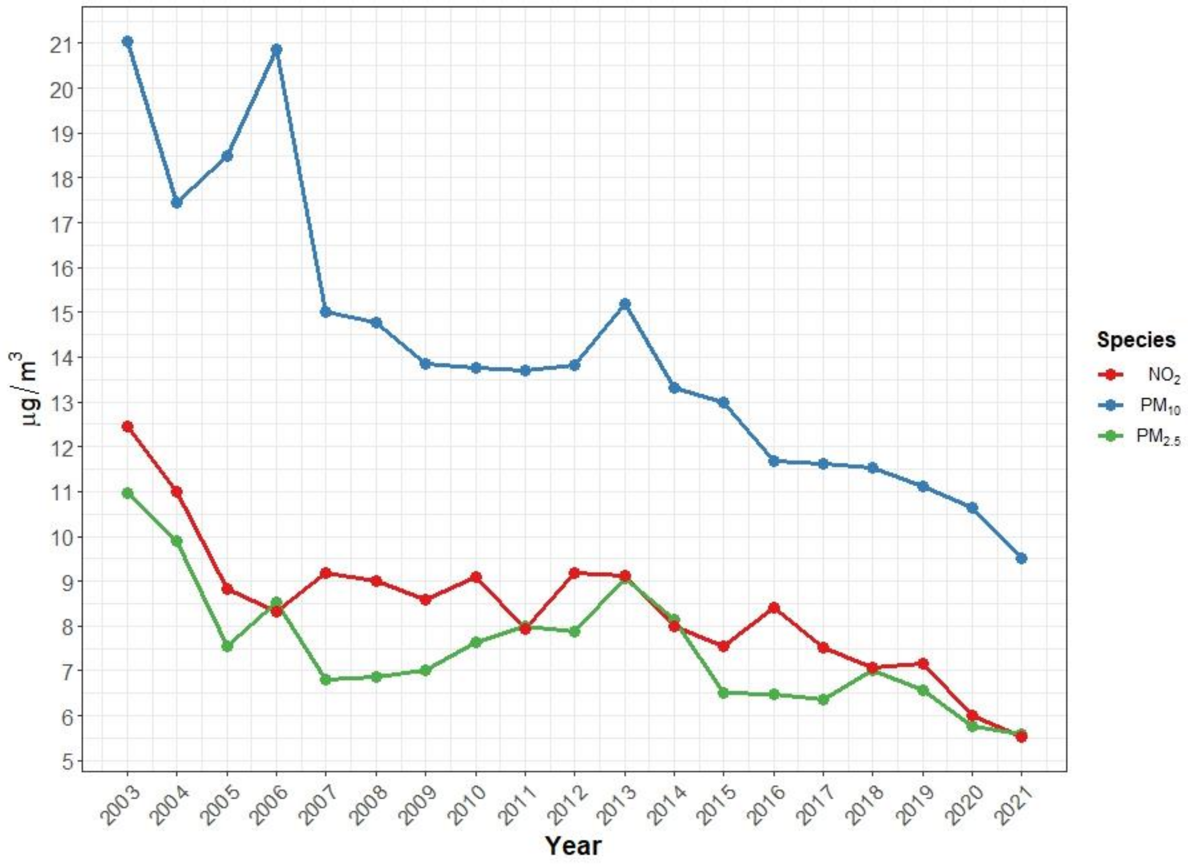

Figure 5: Annual average of entire prediction grid for each pollutant.

Figure S6: Map of contributing NO_2 and PM monitors in 2003 and 2021.

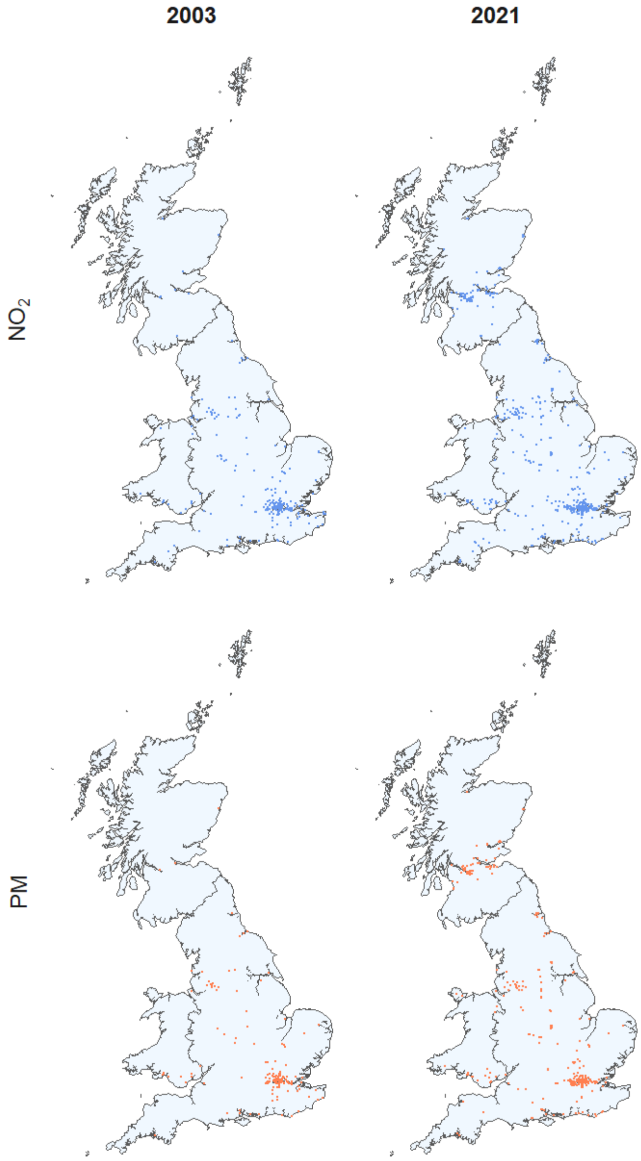

Figure 6: Map of the monitors observing air pollution values in 2003 and 2021 and for each pollutant, NO_2 and PM.

Table S1: Summary of datasets and products used.

Table 1: Summary of source data and datasets used with long name, identifying variable name, spatial and temporal resolution, units, and period when applicable.

Dataset/Product name	Variable (variable id)	Original Spatial Resolution	Original Temporal Resolution	Units	Period	Source Link
VIIRS night-time lights/Annual VNL V1	Night-Light (nightlight)	750m	NA	nanoWatts/cm^2/sr	2015-2016	https://eogdata.mines.edu/products/vnl/ (Elvidge et al., 2017)
UK Gridded Population	Residential and Workday Population Density (respop, workpop)	1km	NA	nPop/km^2	2011	https://catalogue.ceh.ac.uk/documents/0995e94d-6d42-40c1-8ed4-5090d82471e1 (Reis et al., 2017)
Road Traffic Statistics	Road traffic density (trafficmajor, trafficminor)	Road segment	yearly	count points - annual average for daily traffic flow	2003:2021	https://roadtraffic.dft.gov.uk/downloads (DfT, 2023)
CAMS Global Reanalysis (EAC4)	Reanalysis AOD 0.47 (aod47ra)	80km	00:00 and 12:00	dimensionless	2003:2021	https://ads.atmosphere.copernicus.eu/cdsapp#!/dataset/cams-global-reanalysis-eac4?tab=overview (Inness et al., 2019)
	Reanalysis AOD 0.55 (aod55ra)					
	Reanalysis AOD 0.67					
	Reanalysis AOD 0.86					
	Reanalysis AOD 1.24					
	Total Column Nitrogen Dioxide (tcno2ra)			kg/m^2		
European Monitoring and Evaluation Program for the UK (EMEP4UK)	Modelled Surface $PM_{2.5}$ at 50% rel.hum. (EMEP_pm25_rh50)	3km	daily	µg/m^3	2003:2021	https://catalogue.ceh.ac.uk/documents/ca302d30-7b8b-46ec-90b6-67b79df00c92 (Scheffler and Vieno, 2022)
	Modelled Surface PM_{10} at 50% rel.hum. (EMEP_pm10_rh50)					
	Modelled Surface Dust (EMEP_dust)					
	Modelled Surface Coarse Sea Salt (EMEP_seasalt)					
	Modelled Surface SO_2 (EMEP_SO2)					
	Modelled Surface SO_4 (EMEP_SO4)					
	Modelled Surface NO_2 (EMEP_NO2)					
	Modelled Surface NO (EMEP_NO)					

MCD19A2	Satellite AOD 0.47 µm (aod47mod)	1km	daily	NA	2003: 2021	https://lpdaac.usgs.gov/products/mcd19a2v006/ (Lyapustin and Wang, 2018)
	Satellite AOD 0.55 µm (aod55mod)					
L3-OMNO2d	Total Column Nitrogen Dioxide Cloud Cover Screened (tcno2mod)	0.25° x 0.25° (~30km)	daily	Molecules/cm^2	2005: 2021	https://disc.gsfc.nasa.gov/datasets/OMNO2d_003/summary (Krotkov et al., 2013)
DEFRA-Pollution Climate Mapping	Annual Modelled Surface PM_{10} (DEFRA_pm10)	1km	yearly	µg/m^3	2003: 2021	https://uk-air.defra.gov.uk/data/pcm-data (DEFRA, 2023)
	Annual Modelled Surface $PM_{2.5}$5 DEFRA_pm25)					
	Annual Modelled Surface NO_2 (DEFRA_no2)					
AURN, SAQN, WQN, NI, KCL, EU	Air Pollution Monitor Data (NO_2, $PM_{2.5}$, PM_{10})	NA	hourly	µg/m^3	2003: 2021	https://uk-air.defra.gov.uk/networks/ (DEFRA, 2024)
ERA5	Mean Sea Level Pressure (mslp)	0.25° x 0.25° (~30km)	00:00 and 12:00	Pa	2003: 2021	https://cds.climate.copernicus.eu/cdsapp#!/dataset/reanalysis-era5-single-levels?tab=overview (Hersbach et al., 2020)
	Boundary Layer Height (blh00, blh12)			m		
ERA5-Land	Temperature at 2 m (temp2m)	0.1° x 0.1° (~9km)	00:00 and 12:00	K	2003: 2021	https://cds.climate.copernicus.eu/cdsapp#!/dataset/reanalysis-era5-land?tab=overview (Muñoz-Sabater et al., 2021)
	Total Precipitation (precipitation)			m		
	U-Wind component			m/s		
	V-Wind component			m/s		
	Dewpoint temperature at 2m			K		
	Surface pressure			Pa		
Computed from ERA5-Land	Wind Speed (windspeed)	0.1° x 0.1° (~9km)	00:00 and 12:00	m/s	2003: 2021	
	Wind Direction (winddirection)			radians		
	Relative Humidity (relhumidity)			%		
CORINE Land Cover	Nine land classes (lc1, lc2, … lc9)	100m	NA	NA	2012	https://land.copernicus.eu/pan-european/corine-land-cover/clc-2012 (CLMS, 2019)
CLMS EU-DEM v1.1	Elevation (elevation)	25m	NA	m	2011	https://land.copernicus.eu/imagery-in-situ/eu-dem/eu-dem-v1.1 (CLMS, 2016)
IMD	(Imperviousness)	100m	NA	% sealed	2006, 2009, 2012, 2010	https://land.copernicus.eu/pan-european/high-resolution-layers/imperviousness (CLMS, 2020)
MOD13A3	Normalized Difference Vegetation Index (ndvi)	1km	monthly	NDVI (-2000,10000)	2003: 2021	https://lpdaac.usgs.gov/products/mod13a3v061/ (Didan, 2021)

OS OPRoads	Major, Secondary and Local road density (lroadsmajor, lroadsecondary, lroadslocal)	Road segment	NA	segments - metres	2019	https://beta.ordnancesurvey.co.uk/products/os-open-roads (OS, 2023)
UK Airport Data	Inverse distance to airport (d2airport)	NA	NA	metres	2015: 2019	https://www.caa.co.uk/data-and-analysis/uk-aviation-market/airports/uk-airport-data/ (CAA, 2023)
UK Boundary	Inverse distance to sea (d2sea)	NA	NA	metres	NA	https://geoportal.statistics.gov.uk (ONS, 2023a)
Severity of government-imposed safety policies	Stringency Index Simple Average (SIsimpAv)	Nation	daily	NA	2019: 2021	https://github.com/OxCGRT/covid-policy-tracker/blob/master/data/United%20Kingdom/OxCGRT_GBR_latest.csv (Hale et al., 2021)
Coronavirus-19 cases	New Lab-confirmed Cases per Specimen Date (c19cases)	Nation	daily	Number of cases	2019: 2021	https://coronavirus.data.gov.uk/details/download (ONS, 2023b)

Table S2: Observations (days), number of contributing monitors, and yearly mean (ug/m^3) of NO_2, PM_{10}, and $PM_{2.5}$.

Table 2: Yearly summary of retrieved pollutant data, number of observations, number of contributing monitors and mean.

	NO_2			PM_{10}			$PM_{2.5}$		
**Year**	**Obs.**	**Mon.**	**Mean (ug/m^3)**	**Obs.**	**Mon.**	**Mean (ug/m^3)**	**Obs.**	**Mon.**	**Mean (ug/m^3)**
2003	101,697	322	42.04	60,996	179	29.03	3,435	10	16.65
2004	103,968	322	38.14	71,667	209	24.91	3,426	10	14.93
2005	112,733	347	37.69	72,504	217	25.66	4,714	14	14.25
2006	119,377	370	37.86	79,783	238	26.17	5,297	17	14.65
2007	125,175	397	37.21	79,476	238	24.3	5,088	16	13.01
2008	139,012	426	37.29	100,862	301	22.38	9,091	52	12.09
2009	142,418	436	36.78	98,502	302	21.62	23,329	77	12.53
2010	138,769	435	38.18	92,824	284	21.61	26,486	83	13.3
2011	138,375	430	35.2	90,189	272	22.78	25,596	82	14.18
2012	137,768	430	36.54	87,882	263	20.55	27,897	84	12.93
2013	139,315	427	34.87	83,598	252	21.33	26,780	82	13.04
2014	134,480	425	34.37	84,546	258	19.76	28,427	91	12.27
2015	154,133	470	32.01	88,808	268	18.25	34,854	109	10.1
2016	172,965	528	33.6	103,521	309	18.1	50,101	157	10.28
2017	182,779	538	30.97	106,485	316	17.12	55,945	175	9.48
2018	179,341	530	29.09	100,771	296	17.75	57,711	172	9.33
2019	190,214	568	28.33	112,599	337	17.15	66,175	198	9.14
2020	232,436	700	20.33	110,651	323	15.4	68,243	204	7.49
2021	230,039	697	21.87	111,654	326	14.84	74,954	226	7.62
Mean	**151,315**	**463.05**	**33.81**	**91,438**	**273.05**	**20.98**	**31,450**	**97.84**	**11.96**
Total	**2,874,994**			**1,737,318**			**597,549**		

Table S3: Land cover reclassification

Table 3 Grouping of the 44 land classes was carried out to allow for the inclusion of land-cover data in the model. As well as reducing the number of features, grouping land-cover types with rarer, yet similar ones, avoids issues at the prediction stage that would arise from underrepresentation in the training data.

**Original class**	**New class**	**New class id.**
Continuous_urban_fabric	CONTI-URBAN / DISCONT-URBAN / ROADS-RAIL / INDUSTRIAL / COMMERCIAL	lc1urban
Discontinuous_urban_fabric		
Industrial_or_commercial_units		
Road_and_rail_networks_and_associated_land		
Construction_sites		
Airports	AIRPORT	lc2airport
Port_areas	PORT / MINERAL EXTRACTION / DUMP SITES	lc3industr
Mineral_extraction_sites		
Dump_sites		
Green_urban_areas	VEGETATION	lc4vegetation
Sport_and_leisure_facilities		
Agro-forestry_areas		
Broad-leaved_forest		
Coniferous_forest		
Mixed_forest		
Natural_grasslands		
Moors_and_heathland		
Sclerophyllous_vegetation		
Transitional_woodland-shrub		
Sparsely_vegetated_areas		
Non-irrigated_arable_land	AGRICULTURE/ARABLE/CROP	lc5agric
Vineyards		
Fruit_trees_and_berry_plantations		
Olive_groves		
Annual_crops_associated_with_permanent_crops		
Complex_cultivation_patterns		
Land_principally_occupied_by_agriculture_with_significant_areas_of_natural_vegetation		
Pastures	PASTURES	lc6pastures
Permanently_irrigated_land	GREEN + WATER	lc7greenwater

Rice_fields		
Inland_marshes		
Peat_bogs		
Salt_marshes		
Glaciers_and_perpetual_snow	WATER	lc8water
Salines		
Intertidal_flats		
Water_courses		
Water_bodies		
Coastal_lagoons		
Estuaries		
Sea_and_ocean		
Beaches_dunes_sands	NO.URBAN / NO.VEG / NO.WATER (BARE ROCKS, BURNT	lc9bare
Bare_rocks		
Burnt_areas		

Table S4: Yearly hyperparameters for the Light Gradient Boosting Machine algorithm.

Table 4: Fine-tuned yearly hyperparameters used in Stage 1, $PM_{2.5}$ reconstruction from co-located PM_{10} observations. Hyperparameter definitions can be found in the software documentation (Shi et al., 2017).

Stage 1 - LGBM hyperparameters										
	learning_rate	num_leaves	max_depth	min_data_in_leaf	bagging_fraction	bagging_freq	feature_fraction	lambda_l1	lambda_l2	max_bin
2003	0.05702477	3,936	12	10	0.7941480	5	0.5634852	0.5343096	0.9901066	600
2004	0.09902572	2,276	8	20	0.6760309	4	0.5065622	0.5535724	0.6625608	600
2005	0.07221124	1,188	8	10	0.9376545	3	0.5760032	0.8942990	0.6577916	600
2006	0.08962929	3,437	8	6	0.7783517	5	0.5928006	0.5761560	0.9376865	600
2007	0.05660319	262	4	6	0.8426807	3	0.6010858	0.8488871	0.7602141	600
2008	0.06268912	2,722	12	10	0.7090095	3	0.5297345	0.9175661	0.7358471	600
2009	0.06530549	2,052	12	10	0.9134461	5	0.5443306	0.7155563	0.5731831	600
2010	0.05288689	194	12	20	0.9948003	5	0.5752803	0.9012010	0.9693651	600
2011	0.05943258	2,354	12	6	0.9322447	5	0.6525382	0.5682312	0.5424670	600
2012	0.07907751	3,734	12	6	0.8177437	3	0.6621830	0.6013039	0.6477878	600
2013	0.05702477	3,936	12	10	0.7941480	5	0.5634852	0.5343096	0.9901066	600
2014	0.05861061	2,438	12	6	0.8470912	4	0.5374229	0.6773021	0.8047911	600
2015	0.08655718	2,804	12	6	0.9081467	5	0.5173889	0.7863310	0.8509979	60
2016	0.08549376	726	12	6	0.8646732	3	0.5661136	0.6707187	0.5971954	600
2017	0.09016895	1,294	12	10	0.7905904	3	0.5292214	0.5825929	0.6321441	600
2018	0.08549376	726	12	6	0.8646732	3	0.5661136	0.6707187	0.5971954	600
2019	0.09833150	885	12	6	0.8856171	4	0.5938525	0.5539369	0.9510897	600
2020	0.09598678	3,359	12	6	0.6282238	5	0.5488714	0.6761535	0.5735367	600
2021	0.08201795	1,705	12	6	0.8903414	4	0.5596326	0.7885133	0.8646700	600

Table S5: Variables included in stage 3 base-learner models.

Table 5: Variables included in the stage 3 base-learner models. Note Land cover class bare, "lc9bare", was never included. All remaining land-cover classes were always included.

**Variable**	**NO_2**	**PM_{10}**	**$PM_{2.5}$**
DEFRA_PM10		X	X
DEFRA_PM25		X	X
DEFRA_no2	X		
EMEP_NO	X		
EMEP_NO2	X		
EMEP_SO2		X	X
EMEP_SO4		X	X
EMEP_dust		X	X
EMEP_pm10rh50		X	X
EMEP_pm25rh50		X	X
EMEP_seasalt		X	X
SIsimpAv	X	X	X
aod47ra		X	
aod55ra		X	
aod47mod		X	X
aod55mod		X	X
blh00	X	X	X
blh12	X	X	X
c19cases	X	X	X
elevation	X	X	X
imperviousness	X	X	X
d2airport	X	X	X
d2sea	X	X	X
lroadslocal	X	X	X
lroadsmajor	X	X	X
lroadssecondary	X	X	X
mslp	X	X	X
ndvi	X	X	X
nightlight	X	X	X
precipitation	X	X	X
relhumidity	X	X	X
respop	X	X	X
stdelevation	X	X	X
surfpressure	X	X	X
tcno2ra	X		
tcno2mod	X		
temp2m	X	X	X
trafficmajor	X	X	X
trafficminor	X	X	X
winddirection	X	X	X

windspeed	X	X	X
workpop	X	X	X

Table S6: Stage 1, overall, spatial, and temporal performance results of yearly LGBM models.

Table 6: Ten-fold monitor-blocked cross-validated predictions were regressed against observations to obtain R^2, RMSE (ug/m^3), slope (ug/m^3) and intercept (ug/m^3) values. Overall R^2 ranged from 0.661 (2006) to 0.926 (2017) with an average R^2 of 0.80 and RMSE of 3.731 over 19 years.

**Stage 1: $PM_{2.5}$ reconstruction - LGBM performance**												
	Overall				Spatial				Temporal			
	R^2	RMSE	Inter.	Slope	R^2	RMSE	Inter.	Slope	R^2	RMSE	Inter.	Slope
2003	0.667	6.386	1.370	0.908	0.372	4.173	6.925	0.591	0.797	4.456	-0.000	1.072
2004	0.735	4.522	0.289	0.979	0.709	2.634	1.872	0.875	0.756	3.631	-0.000	1.055
2005	0.678	5.617	0.549	0.952	0.563	3.525	3.487	0.770	0.739	4.345	-0.000	1.086
2006	0.661	5.712	2.058	0.848	0.497	4.299	4.663	0.705	0.791	3.573	0.000	1.021
2007	0.735	4.550	-0.823	1.079	0.635	2.507	2.226	0.858	0.748	3.807	-0.000	1.159
2008	0.693	4.740	-0.236	1.034	0.609	4.655	-3.443	1.375	0.779	3.578	0.000	1.123
2009	0.738	4.460	0.608	0.967	0.410	2.742	5.400	0.592	0.834	3.252	0.000	1.070
2010	0.723	4.708	-0.182	1.059	0.502	2.847	3.202	0.800	0.794	3.640	0.000	1.119
2011	0.829	4.439	0.046	1.025	0.351	3.057	6.211	0.577	0.878	3.521	0.000	1.063
2012	0.834	3.838	0.379	0.972	0.417	2.437	4.139	0.688	0.889	2.954	0.000	1.004
2013	0.829	3.755	0.060	1.034	0.487	2.142	4.131	0.710	0.886	2.893	0.000	1.086
2014	0.883	3.100	-0.174	1.046	0.651	1.751	1.430	0.907	0.912	2.546	-0.000	1.062
2015	0.865	2.754	0.189	1.009	0.674	1.455	2.002	0.821	0.894	2.291	0.000	1.041
2016	0.919	2.240	-0.077	1.019	0.850	1.205	0.461	0.966	0.934	1.872	0.000	1.029
2017	0.926	2.067	0.042	1.010	0.849	1.158	0.546	0.957	0.939	1.732	0.000	1.020
2018	0.908	1.993	-0.096	1.023	0.830	1.057	0.301	0.983	0.922	1.700	0.000	1.032
2019	0.923	2.025	0.046	1.010	0.753	1.179	1.024	0.897	0.941	1.678	0.000	1.020
2020	0.897	1.886	0.010	1.022	0.815	0.985	0.600	0.939	0.912	1.615	0.000	1.035
2021	0.838	2.106	0.023	1.020	0.782	1.011	0.693	0.927	0.849	1.860	-0.000	1.038
**Mean**	**0.804**	**3.732**	**0.215**	**1.001**	**0.619**	**2.359**	**2.414**	**0.839**	**0.852**	**2.892**	**0.000**	**1.060**

Table S7: Stage 2, overall model performance results for satellite data reconstruction.

Table7: Stage 2, overall performance statistics. NO_2 column reconstruction showed good performance (R^2 ranging between 0.739 and 0.840) and a persistent but small bias in the intercept and slope. AOD reconstruction models, for both 0.47nm and 0.55nm wavelengths, displayed very high performance (R^2 between 0.941 and 0.976) and little to no bias. NO_2 column data was not available for 2003 and 2004.

Stage 2: Satellite data reconstruction - Overall RF performance

	L3-OMNO2d - NO_2				MCD19A2 - AOD 0.47nm				MCD19A2 - AOD 0.55nm			
	R^2	RMSE (molec./cm^2)	Inter.	Slope	R^2	RMSE (dimensionless)	Inter.	Slope	R^2	RMSE (dimensionless)	Inter.	Slope
2003					0.976	0.023	-0.001	1.004	0.976	0.016	-0.000	1.004
2004					0.952	0.027	-0.001	1.008	0.952	0.019	-0.001	1.008
2005	0.796	1.787	-0.476	1.116	0.958	0.025	-0.001	1.007	0.957	0.018	-0.001	1.007
2006	0.792	1.845	-0.472	1.116	0.972	0.024	-0.000	1.004	0.972	0.017	-0.000	1.005
2007	0.833	1.685	-0.366	1.089	0.975	0.024	-0.000	1.004	0.975	0.017	-0.000	1.004
2008	0.829	1.579	-0.390	1.105	0.956	0.025	-0.001	1.007	0.955	0.018	-0.001	1.008
2009	0.814	1.364	-0.389	1.121	0.959	0.025	-0.001	1.007	0.959	0.018	-0.001	1.007
2010	0.812	1.481	-0.409	1.121	0.947	0.025	-0.001	1.009	0.947	0.018	-0.001	1.009
2011	0.840	1.411	-0.373	1.106	0.973	0.023	-0.001	1.005	0.973	0.016	-0.000	1.005
2012	0.830	1.320	-0.350	1.108	0.960	0.024	-0.001	1.007	0.960	0.017	-0.001	1.007
2013	0.818	1.239	-0.375	1.123	0.963	0.025	-0.001	1.006	0.963	0.018	-0.000	1.006
2014	0.799	1.237	-0.421	1.140	0.947	0.026	-0.001	1.008	0.947	0.018	-0.001	1.008
2015	0.816	1.124	-0.373	1.128	0.945	0.025	-0.001	1.009	0.945	0.018	-0.001	1.009
2016	0.822	1.137	-0.381	1.128	0.950	0.025	-0.001	1.007	0.950	0.018	-0.001	1.008
2017	0.805	1.031	-0.388	1.149	0.942	0.025	-0.001	1.009	0.941	0.018	-0.001	1.010
2018	0.798	0.976	-0.361	1.143	0.953	0.023	-0.001	1.008	0.953	0.016	-0.001	1.008
2019	0.823	0.957	-0.337	1.127	0.958	0.024	-0.001	1.007	0.958	0.017	-0.001	1.007
2020	0.750	0.869	-0.412	1.185	0.955	0.023	-0.001	1.008	0.955	0.016	-0.001	1.008
2021	0.739	0.980	-0.453	1.195	0.956	0.024	-0.001	1.007	0.956	0.017	-0.001	1.007
**Mean**	**0.807**	**1.295**	**-0.396**	**1.129**	**0.958**	**0.024**	**-0.001**	**1.007**	**0.958**	**0.017**	**-0.001**	**1.007**

Table S8: Top 15 features by average relative importance by ML algorithm and pollutant modelled.

Green: EMEP and DEFRA Orange: Spatial lag Blue: Atmospheric reanalysis

Table 8: Fifteen most important variables by pollutant and base-learner. As tree-based algorithms, ranger, extreme gradient boosting, and light gradient boosting machine compute measures of importance based on information gain calculated at the splitting of each variable. Variable belonging to the EMEP dataset are shown in green, those referred to as spatial lag are shown in orange, and those derived from atmospheric reanalysis are shown in blue.

	no2rang	no2xgb	no2lgb	pm10rang	pm10xgb	pm10lgb	pm25rang	pm25xgb	pm25lgb
1	EMEP_NO2	EMEP_NO2	EMEP_NO2	EMEP_pm25rh50	EMEP_pm25rh50	EMEP_pm25rh50	EMEP_pm25rh50	EMEP_pm25rh50	EMEP_pm25rh50
2	EMEP_NO	EMEP_NO	d2hs	EMEP_pm10rh50	EMEP_pm10rh50	EMEP_pm10rh50	EMEP_pm10rh50	EMEP_pm10rh50	EMEP_pm10rh50
3	d2hs	d2hs	EMEP_NO	EMEP_SO4	EMEP_SO4	doy	EMEP_SO4	EMEP_SO4	doy
4	DEFRA_no2	DEFRA_no2	respop	precipitation	winddirection	precipitation	winddirection	doy	EMEP_SO4
5	d2bkg	respop	DEFRA_no2	winddirection	precipitation	winddirection	blh00	winddirection	idw1background
6	workpop	d2bkg	temp2m	mslp	doy	mslp	precipitation	blh00	winddirection
7	idw2background	workpop	doy	blh00	mslp	idw1background	idw1background	precipitation	idw2hotspot
8	idw1background	temp2m	windspeed	doy	blh00	EMEP_SO4	idw2background	mslp	precipitation
9	respop	idw1background	idw2hotspot	EMEP_dust	EMEP_dust	EMEP_dust	idw2hotspot	idw1background	blh00
10	idw1hotspot	windspeed	d2bkg	EMEP_SO2	temp2m	blh00	doy	idw2background	idw2background
11	idw2hotspot	idw1hotspot	workpop	idw1background	idw1background	d2hs	EMEP_SO2	idw2hotspot	mslp
12	temp2m	idw2background	idw1hotspot	idw1hotspot	d2hs	idw1hotspot	mslp	EMEP_seasalt	EMEP_seasalt
13	windspeed	doy	idw2background	idw2hotspot	EMEP_seasalt	idw2hotspot	EMEP_seasalt	EMEP_dust	EMEP_dust
14	nightlight	idw2hotspot	idw1background	temp2m	idw1hotspot	EMEP_seasalt	EMEP_dust	idw1hotspot	temp2m
15	imperviousness	blh00	blh00	EMEP_seasalt	idw2hotspot	temp2m	idw1hotspot	blh12	idw1hotspot

Table S9: Total average spatial and temporal performance

Table 9: Full-period (2003-2021) average of spatial and temporal R^2, RMSE (μg/m³), slope (μg/m³), and intercept (μg/m³) for each base learner and ensemble learner.
All temporal intercept values are shown as blank as they are 0 by default.

		Stage 3 - Base and ensemble learner spatial and temporal cross-validated performance							
		Spatial				Temporal			
		R^2	RMSE	Inter.	Slope	R^2	RMSE	Inter.	Slope
NO_2	RF	0.665	9.472	2.670	0.993	0.691	8.286	---	1.093
	XGB	0.664	9.472	2.428	1.001	0.701	8.126	---	1.031
	LGBM	0.666	9.452	2.445	0.989	0.679	8.423	---	1.018
	RIDGE	0.520	11.311	6.173	0.895	0.486	10.690	---	0.924
	LASSO	0.510	11.428	6.625	0.874	0.490	10.648	---	0.902
	NNLS	0.665	9.471	2.513	0.985	0.707	8.053	---	1.040
PM_{10}	RF	0.504	3.794	1.517	0.972	0.751	5.126	---	1.065
	XGB	0.498	3.816	1.880	0.958	0.761	5.023	---	1.048
	LGBM	0.496	3.824	1.774	0.955	0.750	5.137	---	1.033
	RIDGE	0.423	4.092	2.350	0.964	0.476	7.416	---	1.194
	LASSO	0.423	4.091	2.713	0.942	0.482	7.372	---	1.134
	NNLS	0.497	3.820	1.828	0.951	0.763	5.006	---	1.045
$PM_{2.5}$	RF	0.646	1.862	0.692	0.979	0.853	2.560	---	1.059
	XGB	0.639	1.878	0.974	0.954	0.858	2.515	---	1.031
	LGBM	0.642	1.870	0.834	0.963	0.853	2.564	---	1.034
	RIDGE	0.578	2.036	0.923	0.997	0.575	4.360	---	1.222
	LASSO	0.574	2.044	1.268	0.960	0.583	4.321	---	1.167
	NNLS	0.641	1.872	0.891	0.956	0.861	2.484	---	1.034

Table S10: Summaries of predictor values at monitor locations.

Table 10 Summary values (minimum, maximum, mean, standard deviation and median) of variables at monitor locations. Values are the full period average.

Variable	Min.	Max.	Mean	S.D.	Median
**DEFRA_PM10**	7.709	24.528	16.679	3.466	16.777
**DEFRA_PM25**	4.769	16.761	11.239	2.471	11.477
**DEFRA_no2**	2.915	53.257	22.44	9.436	21.094
**EMEP_NO**	0.016	370.886	6.852	16.587	2.308
**EMEP_NO2**	0.383	86.1	20.044	13.507	16.608
**EMEP_SO2**	0.012	226.773	4.788	6.277	3.015
**EMEP_SO4**	0.083	39.225	1.985	1.953	1.384
**EMEP_dust**	0	18.335	0.308	0.878	0.051
**EMEP_pm10rh50**	1.386	167.137	20.256	11.91	17.539
**EMEP_pm25rh50**	0.877	124.198	12.738	10.501	9.231
**EMEP_seasalt**	0.007	41.025	4.561	4.448	2.99
**SIsimpAv**	1.218	9.259	5.619	2.661	6.189
**aod47mod**	0.009	1.627	0.285	0.136	0.285
**aod47ra**	0.009	1.038	0.186	0.119	0.162
**aod55mod**	0.006	1.245	0.2	0.098	0.2
**aod55ra**	0.007	0.874	0.158	0.098	0.139
**blh00**	10.721	2183.243	448.478	343.147	367.932
**blh12**	30.149	2431.663	936.009	393.715	919.993
**c19cases**	1.105	15688.63	1315.357	1890.988	599.053
**d2airport**	0.689	224.585	27.557	26.591	18.032
**d2sea**	0.071	120.094	41.162	29.546	41.331
**elevation**	1.145	672.885	50.886	58.702	31.95
**imperviousness**	0.027	90.235	42.957	20.878	43.872
**lc1urban**	0	1	0.756	0.307	0.89
**lc2airport**	0	1	0.019	0.114	0
**lc3industr**	0	0.537	0.011	0.059	0
**lc4vegetation**	0	0.995	0.081	0.161	0
**lc5agric**	0	1	0.055	0.157	0
**lc6pastures**	0	0.951	0.056	0.149	0
**lc7greenwater**	0	0.603	0.006	0.05	0
**lc8water**	0	0.557	0.015	0.066	0
**lc9bare**	0	0.153	0	0.008	0
**lroadslocal**	0	24.001	11.233	4.629	11.526
**lroadsmajor**	0	6.865	1.857	1.401	1.608
**lroadssecondary**	0	3.181	0.458	0.637	0.048
**mslp**	96665.69	104283.3	101474.1	1098.279	101573.2
**ndvi**	3992365	87561661	47892452	13511435	48626660
**nightlight**	0	141.146	36.046	28.212	28.693
**precipitation**	0	0.033	0.002	0.002	0.001
**relhumidity**	45.967	99.579	78.848	9.331	79.351
**respop**	0	22620	4499.903	3700.249	3776.789

**stdelevation**	0.682	90.734	7.471	8.501	5.157
**surfpressure**	93412.95	104107.5	100561.4	1285.515	100696.5
**tcno2mod**	0.016	38.612	5.21	3.348	4.575
**tcno2ra**	0	0	0	0	0
**temp2m**	267.331	297.641	283.621	5.195	283.62
**trafficmajor**	0	539935.7	55951.31	74363.39	31289.87
**trafficminor**	0	44239.25	4206.791	6605.654	302.881
**winddirection**	0.004	359.993	200.088	90.061	220.519
**windspeed**	0.014	16.567	3.782	1.872	3.572
**workpop**	0	119894.5	7959.392	13301.41	4398.631

256

Table S11: Summaries of variables over prediction grid

Table 11: Summary values (minimum, maximum, mean, standard deviation and median) of variables throughout entire target grid. Values are the full period average.

Variable	Min.	Max.	Mean	S.D.	Median
**DEFRA_PM10**	5.727	25.659	11.451	3.597	11.233
**DEFRA_PM25**	3.493	17.88	7.353	2.447	7.156
**DEFRA_no2**	1.02	53.739	7.144	5.124	5.786
**EMEP_NO**	0	386.658	1.039	3.98	0.272
**EMEP_NO2**	0.045	93.819	6.472	7.492	3.784
**EMEP_SO2**	0.001	412.437	1.609	2.666	0.807
**EMEP_SO4**	0.027	70.515	1.404	1.476	0.935
**EMEP_dust**	0	106.569	0.253	0.828	0.034
**EMEP_pm10rh50**	0.62	215.601	14.815	9.163	12.712
**EMEP_pm25rh50**	0.511	176.229	8.246	8.056	5.194
**EMEP_seasalt**	0.002	59.644	5.062	4.923	3.376
**aod47mod**	0	3.008	0.278	0.131	0.275
**aod47ra**	0.005	1.093	0.172	0.113	0.15
**aod55mod**	0	2.343	0.195	0.094	0.192
**aod55ra**	0.004	0.921	0.148	0.094	0.13
**blh00**	10.008	2502.711	494.767	365.093	425.134
**blh12**	14.699	2570.506	895.051	377.81	877.622
**d2airport**	0.175	414.503	78.648	59.946	63.588
**d2sea**	0	127.685	31.221	28.54	23.659
**elevation**	-2.559	1182.733	154.362	149.177	105.812
**imperviousness**	0	91.196	3.205	8.807	0.329
**invd2airport**	0.002	5.853	0.024	0.047	0.016
**invd2sea**	0.008	452152	3.372	892.513	0.045
**lc1urban**	0	1	0.065	0.193	0
**lc2airport**	0	1	0.001	0.028	0
**lc3industr**	0	1	0.003	0.033	0
**lc4vegetation**	0	1	0.247	0.348	0.018
**lc5agric**	0	1	0.265	0.365	0
**lc6pastures**	0	1	0.266	0.34	0.06
**lc7greenwater**	0	1	0.092	0.251	0
**lc8water**	0	1	0.059	0.204	0
**lc9bare**	0	1	0.001	0.024	0
**lroadslocal**	0	34.813	1.886	2.698	1.199
**lroadsmajor**	0	8.82	0.21	0.527	0
**lroadssecondary**	0	4.401	0.118	0.339	0
**mslp**	96250.79	104337.7	101365.2	1175.36	101476.5
**ndvi**	-2E+07	98684213	63357078	14764442	65459220
**nightlight**	0	315.763	1.719	6.351	0.006
**precipitation**	0	0.04	0.002	0.003	0.001
**relhumidity**	42.763	99.942	80.654	8.937	81.23
**respop**	0	22620	69.434	500.346	0

**stdelevation**	0	238.14	18.565	20.707	11.495
**surfpressure**	89227.59	104293.9	99571.94	1866.024	99864.01
**tcno2mod**	0.001	41.495	3.33	2.702	2.453
**tcno2ra**	0	0	0	0	0
**temp2m**	263.967	297.884	282.58	4.992	282.581
**trafficmajor**	0	1390257	4405.799	20748.36	0
**trafficminor**	0	67881.84	661.139	2404.44	0
**winddirection**	0	360	202.298	87.551	218.73
**windspeed**	0	20.607	3.999	2.197	3.67
**workpop**	0	126203	69.434	633.684	0

References

CAA, (Civil Aviation Authority), 2023. Airport Data. https://www.caa.co.uk/data-and-analysis/uk-aviation-market/airports/uk-airport-data/, accessed in 2023.

CLMS, (Copernicus Land Monitoring Service), 2016. European Digital Elevation Model (EU-DEM), version 1.1.

CLMS, (Copernicus Land Monitoring Service), 2019. Corine Land Cover (CLC) 2018, Version 2020_20u1.

CLMS, (Copernicus Land Monitoring Service), 2020. Imperviousness Density 2018.

Copernicus Knowledge Base, 2023. ERA5: How to calculate wind speed and wind direction from u and v components of the wind? ECMWF Conflu. Wiki. https://confluence.ecmwf.int/pages/viewpage.action?pageId=133262398, accessed in 2023.

DEFRA, (Department for Environment Food and Rural Affairs), 2023. Modelled background pollution data.

DEFRA, (Department for Environment Food and Rural Affairs), 2024. Openair data definitions- Defra, UK. https://uk-air.defra.gov.uk/data/openair-data-definition, accessed in 2023.

DfT, (Department for Transport), 2023. Road traffic statistics - AADF Data - major and minor roads.

Didan, K., 2021. MODIS/Terra Vegetation Indices Monthly L3 Global 1km SIN Grid V061. https://doi.org/10.5067/MODIS/MOD13A3.061

Elvidge, C.D., Baugh, K., Zhizhin, M., Hsu, F.C., Ghosh, T., 2017. VIIRS night-time lights. Int. J. Remote Sens. 38, 5860–5879.

Hale, T., Angrist, N., Goldszmidt, R., Kira, B., Petherick, A., Phillips, T., Webster, S., Cameron-Blake, E., Hallas, L., Majumdar, S., Tatlow, H., 2021. A global panel database of pandemic policies (Oxford COVID-19 Government Response Tracker). Nat. Hum. Behav. 5, 529–538. https://doi.org/10.1038/s41562-021-01079-8

Hersbach, H., Bell, B., Berrisford, P., Hirahara, S., Horányi, A., Muñoz-Sabater, J., Nicolas, J., Peubey, C., Radu, R., Schepers, D., Simmons, A., Soci, C., Abdalla, S., Abellan, X., Balsamo, G., Bechtold, P., Biavati, G., Bidlot, J., Bonavita, M., De Chiara, G., Dahlgren, P., Dee, D., Diamantakis, M., Dragani, R., Flemming, J., Forbes, R., Fuentes, M., Geer, A., Haimberger, L., Healy, S., Hogan, R.J., Hólm, E., Janisková, M., Keeley, S., Laloyaux, P., Lopez, P., Lupu, C., Radnoti, G., de Rosnay, P., Rozum, I., Vamborg, F., Villaume, S., Thépaut, J.-N., 2020. The ERA5 global reanalysis. Q. J. R. Meteorol. Soc. 146, 1999–2049. https://doi.org/10.1002/qj.3803

Inness, A., Ades, M., Agustí-Panareda, A., Barré, J., Benedictow, A., Blechschmidt, A.-M., Dominguez, J.J., Engelen, R., Eskes, H., Flemming, J., Huijnen, V., Jones, L., Kipling, Z., Massart, S., Parrington, M., Peuch, V.-H., Razinger, M., Remy, S., Schulz, M., Suttie, M., 2019. The CAMS reanalysis of atmospheric composition. Atmospheric Chem. Phys. 19, 3515–3556. https://doi.org/10.5194/acp-19-3515-2019

Krotkov, N.A., Lok, L.N., Marchenko, S.V., Celarier, E.A., Bucsela, E.J., Swartz, W.H., Joiner, J., 2013. OMI/Aura NO2 Cloud-Screened Total and Tropospheric Column Daily L3 Global 0.25deg Lat/Lon Grid. https://doi.org/10.5067/AURA/OMI/DATA3007

Lyapustin, A., Wang, Y., 2018. MCD19A2 MODIS/Terra+Aqua Land Aerosol Optical Depth Daily L2G Global 1km SIN Grid V006. https://doi.org/10.5067/MODIS/MCD19A2.006

Muñoz-Sabater, J., Dutra, E., Agustí-Panareda, A., Albergel, C., Arduini, G., Balsamo, G., Boussetta, S., Choulga, M., Harrigan, S., Hersbach, H., Martens, B., Miralles, D.G., Piles, M., Rodríguez-Fernández, N.J., Zsoter, E., Buontempo, C., Thépaut, J.-N., 2021. ERA5-Land: a state-of-the-art global reanalysis dataset for land applications. Earth Syst. Sci. Data 13, 4349–4383. https://doi.org/10.5194/essd-13-4349-2021

National Physics Laboratory, 2023. How do I convert between dew point and relative humidity? NPLWebsite. https://www.npl.co.uk/resources/q-a/dew-point-and-relative-humidity, accessed in 2023.
ONS, (Office for National Statistics), 2023a. Open Geography Portal. https://geoportal.statistics.gov.uk/, accessed in 2023.
ONS, (Office for National Statistics), 2023b. Coronavirus in the UK | newCasesBySpecimenDate.
OS, (Ordnance Survey), 2023. OS Open Roads.
Reis, S., Liska, T., Steinle, S., Carnell, E., Leaver, D., Roberts, E., Vieno, M., Beck, R., Dragosits, U., 2017. UK gridded population 2011 based on Census 2011 and Land Cover Map 2015. https://doi.org/10.5285/0995e94d-6d42-40c1-8ed4-5090d82471e1
Scheffler, J., Vieno, M., 2022. European Monitoring and Evaluation Program Model for the UK (EMEP4UK) daily atmospheric composition for 2002-2021. https://doi.org/10.5285/ca302d30-7b8b-46ec-90b6-67b79df00c92
Schneider, R., Vicedo-Cabrera, A., Sera, F., Masselot, P., Stafoggia, M., de Hoogh, K., Kloog, I., Reis, S., Vieno, M., Gasparrini, A., 2020. A Satellite-Based Spatio-Temporal Machine Learning Model to Reconstruct Daily PM2.5 Concentrations across Great Britain. Remote Sens. 12, 3803. https://doi.org/10.3390/rs12223803
Shi, Y., Ke, G., Soukhavong, D., Lamb, J., 2017. LightGBM Docs: Parameters.
Wright, M.N., Ziegler, A., 2017. ranger: A Fast Implementation of Random Forests for High Dimensional Data in C++ and R. J. Stat. Softw. 77, 1–17. https://doi.org/10.18637/jss.v077.i01
